# Correction: Specific blockade of Rictor-mTOR association inhibits mTORC2 activity and is cytotoxic in glioblastoma

**DOI:** 10.1371/journal.pone.0212160

**Published:** 2019-02-06

**Authors:** Angelica Benavides-Serrato, Jihye Lee, Brent Holmes, Kenna A. Landon, Tariq Bashir, Michael E. Jung, Alan Lichtenstein, Joseph Gera

The structures of the JR-AB2 series of compounds reported are incorrect and their correct structures have now been assigned. Thus JR-AB2-000, also called CID613034, which was obtained from the Developmental Therapeutics Program repository at the NCI, does not have the structure given by that organization (and shown in our paper) but rather the structure (new-JR-AB2-000). This was confirmed by single crystal x-ray structure determination. In addition, several other compounds in this series were examined by x-ray crystallography and shown to have analogous structures. Thus, the reaction of the 2-arylamino-4,5-dihydrothiazole with an aryl isocyanate afforded the 2-iminothiazolidine (carbamoylation of the internal nitrogen) and not the (4,5-dihydro-1,3-thiazol-2-yl)urea (carbamoylation of the external nitrogen). Therefore, all of the structures of this series of compounds are the 2-iminothiazolidine isomers. Please see the corrected Fig 1 and Fig 4 here. Please view the correct S4 Fig and S1 File below.

**Fig 1:**
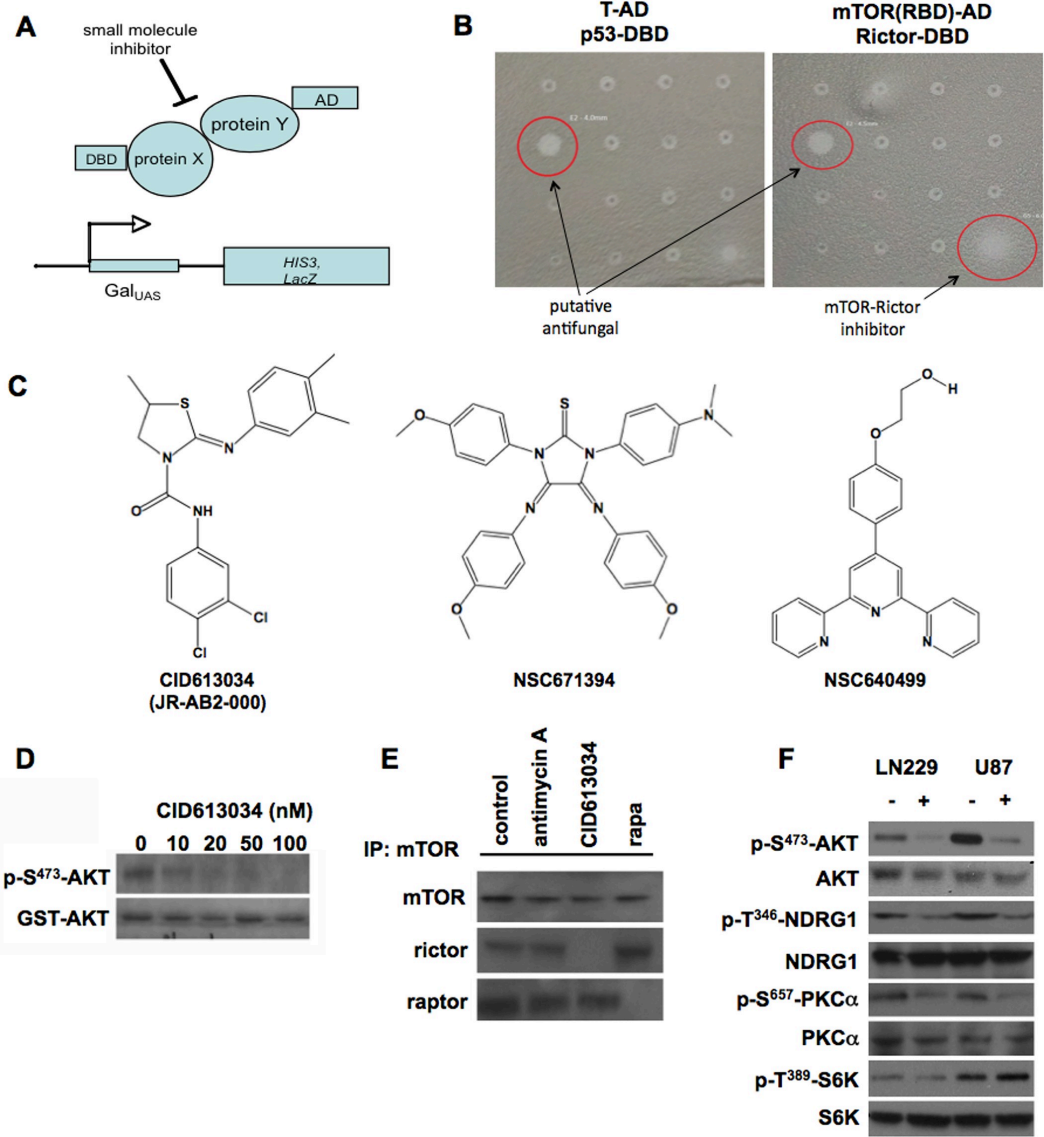
Identification of compounds which inhibit mTORC2 activity in glioblastoma cells. (A) Yeast two-hybrid assay configuration used to screen for inhibitors of the human Rictor-mTOR interaction. Full-length human Rictor fused to the Gal4-DNA-binding domain and mTOR fused to the Gal4-activation domain was expressed in yeast containing reporters harboring *Gal4* upstream activating sequences (UAS). (B) Screening of compounds which block Rictor/mTOR association. Yeast expressing either p53-DBD and SV40 large T antigen-AD fusions or Rictor-DBD and mTOR-AD fusions were plated. Compounds were pinned onto the plate surfaces and examined for halo formation (red circles). Compounds which inhibited Rictor/mTOR-mediated growth on selective media while having little or no effects on p53/T antigen-mediated growth were considered specific. Compounds which blocked growth of both strains were considered nonspecific and exhibited general antifungal activity. (C) Structures of compounds which inhibit Rictor/mTOR association. (D) CID613034 inhibits mTORC2 *in vitro* kinase activity. mTORC2 kinase reactions were performed using GST-tagged AKT as a substrate with the indicated concentrations of inhibitor. Reactions were subsequently immunoblotted for phospho-S^473^-AKT and GST-tagged AKT. (E) CID613034 blocks binding of Rictor to mTOR in LN229 cells. Cells were treated with 5 mM Antimycin (control compound), 50 nM CID613034 or 20 nM rapamycin for 15 min and mTOR immunoprecipitated. Immunoprecipitations were then immunoblotted for the indicated proteins. (F) mTORC2 signaling is inhibited in GBM lines following 24 h exposure to CID613034. LN229 or U87 cells were treated with 100 nM of inhibitor as shown and lysates immunoblotted for the indicated proteins. Data shown are representative of experiments repeated two times.

**Fig 4:**
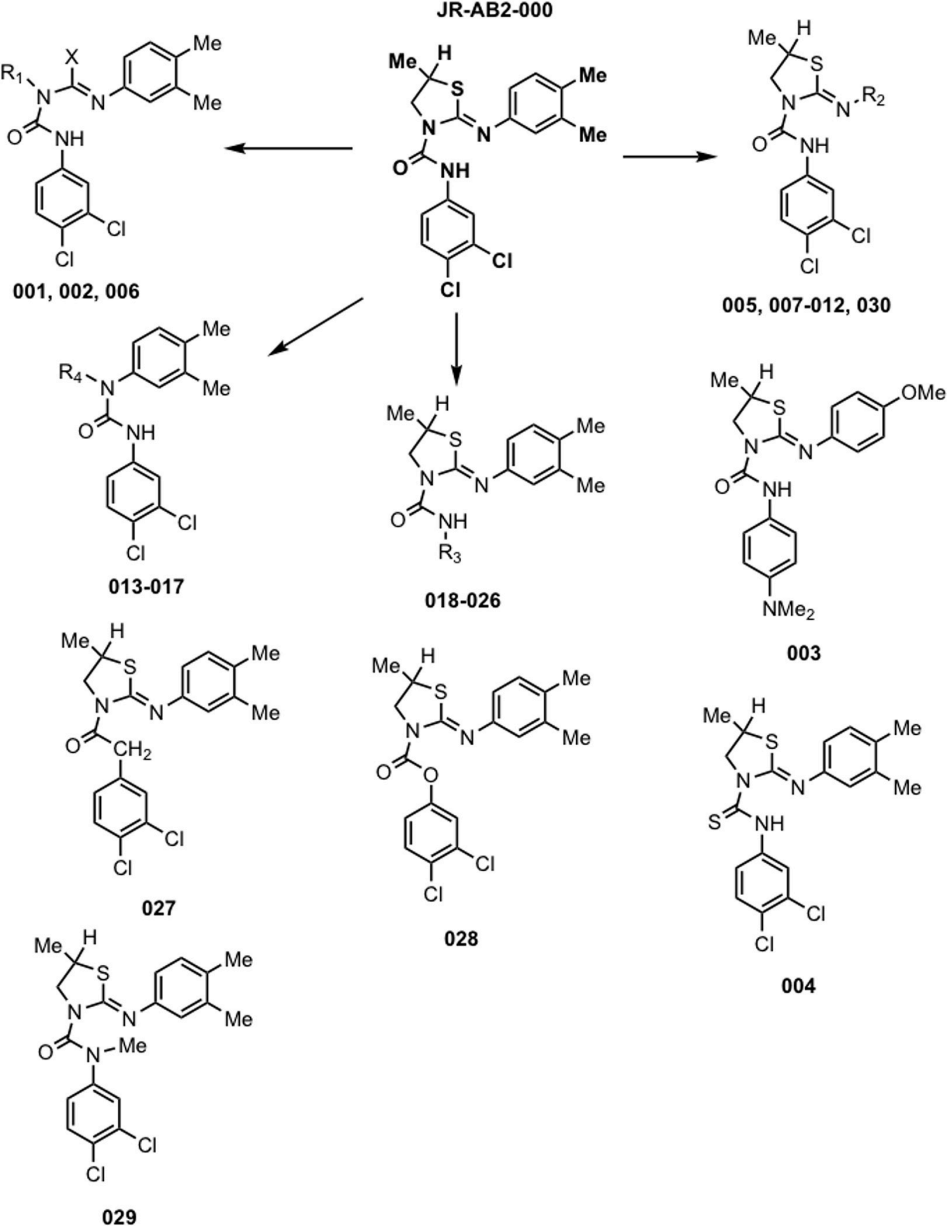
Synthesis of JR-AB2-000 (CID613034) analogs. Thirty analogs were synthesized with the indicated group modification shown and further detailed in Table 1. Analog identification numbers are shown below the structures corresponding to analogs where a particular functional group is modified. Detailed synthetic procedures and NMR spectra are described in supplementary Materials and methods.

## Supporting information

S4 Fig Chemical structures of CID613034 analog modifications. (DOCX)

S1 File Supplemental materials and methods. (DOCX)
