# Supplementary material for: Correction: Specific blockade of Rictor-mTOR association inhibits mTORC2 activity and is cytotoxic in glioblastoma
Source: PLoS One. 2019 Feb 6;14(2):e0212160. doi: 10.1371/journal.pone.0212160 (PMC6364936; doi:10.1371/journal.pone.0212160)
Supplement: S4 Fig — (DOCX) [file pone.0212160.s001.docx]

| Compound | type | R^1^ | R^2^ | R^3^ | R^4^ | R^5^ | X | Y | Z |
| --- | --- | --- | --- | --- | --- | --- | --- | --- | --- |
| JR-AB2-000 | **A** | Me | H | 3,4-Me_2_C_6_H_3_ | 3,4-Cl_2_C_6_H_3_ | H | S | O | N |
| JR-AB2-001 | **A** | H | H | 3,4-Me_2_C_6_H_3_ | 3,4-Cl_2_C_6_H_3_ | H | S | O | N |
| JR-AB2-002 | **A** | Me | Me | 3,4-Me_2_C_6_H_3_ | 3,4-Cl_2_C_6_H_3_ | H | S | O | N |
| JR-AB2-003 | **A** | Me | H | 4-MeOC_6_H_4_ | 4-Me_2_NC_6_H_4_ | H | S | O | N |
| JR-AB2-004 | **A** | Me | H | 3,4-Me_2_C_6_H_3_ | 3,4-Cl_2_C_6_H_3_ | H | S | S | N |
| JR-AB2-005 | **A** | Me | H | 4-MeOC_6_H_4_ | 3,4-Cl_2_C_6_H_3_ | H | S | O | N |
| JR-AB2-006 | **A** | Me | H | 3,4-Me_2_C_6_H_3_ | 3,4-Cl_2_C_6_H_3_ | H | O | O | N |
| JR-AB2-007 | **A** | Me | H | C_6_H_5_ | 3,4-Cl_2_C_6_H_3_ | H | S | O | N |
| JR-AB2-008 | **A** | Me | H | 4-Me_2_NC_6_H_4_ | 3,4-Cl_2_C_6_H_3_ | H | S | O | N |
| JR-AB2-009 | **A** | Me | H | 4-MeC_6_H_4_ | 3,4-Cl_2_C_6_H_3_ | H | S | O | N |
| JR-AB2-010 | **A** | Me | H | 3-MeC_6_H_4_ | 3,4-Cl_2_C_6_H_3_ | H | S | O | N |
| JR-AB2-011 | **A** | Me | H | 3-FC_6_H_4_ | 3,4-Cl_2_C_6_H_3_ | H | S | O | N |
| JR-AB2-012 | **A** | Me | H | Et | 3,4-Cl_2_C_6_H_3_ | H | S | O | N |
| JR-AB2-013 | **B** |  |  | 2-thiazolyl |  |  |  |  |  |
| JR-AB2-014 | **B** |  |  | 2-benzothiazolyl |  |  |  |  |  |
| JR-AB2-015 | **B** |  |  | 4,4-Me_2_-2-thiazolidinyl |  |  |  |  |  |
| JR-AB2-016 | **B** |  |  | 2-pyridyl |  |  |  |  |  |
| JR-AB2-017 | **B** |  |  | C_6_H_5_ |  |  |  |  |  |
| JR-AB2-018 | **A** | Me | H | 3,4-Me_2_C_6_H_3_ | C_6_H_5_ | H | S | O | N |
| JR-AB2-019 | **A** | Me | H | 3,4-Me_2_C_6_H_3_ | 3-ClC_6_H_4_ | H | S | O | N |
| JR-AB2-020 | **A** | Me | H | 3,4-Me_2_C_6_H_3_ | 4-ClC_6_H_4_ | H | S | O | N |
| JR-AB2-021 | **A** | Me | H | 3,4-Me_2_C_6_H_3_ | 4-FC_6_H_4_ | H | S | O | N |
| JR-AB2-022 | **A** | Me | H | 3,4-Me_2_C_6_H_3_ | 4-MeC_6_H_4_ | H | S | O | N |
| JR-AB2-023 | **A** | Me | H | 3,4-Me_2_C_6_H_3_ | 4-MeOC_6_H_4_ | H | S | O | N |
| JR-AB2-024 | **A** | Me | H | 3,4-Me_2_C_6_H_3_ | 1-naphthyl | H | S | O | N |
| JR-AB2-025 | **A** | Me | H | 3,4-Me_2_C_6_H_3_ | Et | H | S | O | N |
| JR-AB2-026 | **A** | Me | H | 3,4-Me_2_C_6_H_3_ | tert-butyl | H | S | O | N |
| JR-AB2-027 | **A** | Me | H | 3,4-Me_2_C_6_H_3_ | 3,4-Cl_2_C_6_H_3_ | H | S | O | CH_2_ |
| JR-AB2-028 | **A** | Me | H | 3,4-Me_2_C_6_H_3_ | 3,4-Cl_2_C_6_H_3_ | H | S | O | O |
| JR-AB2-029 | **A** | Me | H | 3,4-Me_2_C_6_H_3_ | 3,4-Cl_2_C_6_H_3_ | Me | S | O | N |
| JR-AB2-030 | **A** | Me | H | 4-BrC_6_H_4_ | 3,4-Cl_2_C_6_H_3_ | H | S | O | N |
