# Supplementary material for: Correction: Specific blockade of Rictor-mTOR association inhibits mTORC2 activity and is cytotoxic in glioblastoma
Source: PLoS One. 2019 Feb 6;14(2):e0212160. doi: 10.1371/journal.pone.0212160 (PMC6364936; doi:10.1371/journal.pone.0212160)
Supplement: S1 File — (DOCX) [file pone.0212160.s002.docx]

**Supplemental Materials and Methods**

**Antibodies:** Antibodies were from the following sources. mTOR (#2972, Cell Signaling Technologies, CST), phospho-S^312^-IRS1 (#ab66154, Abcam), total IRS1 (#ab131487, Abcam), phospho-T^202/Y204^-ERK (#4370, CST), total ERK (#9102, CST), phospho-S^657^-PKCα (#SAB-4504096, Sigma), total PKCα (#sc-208, Santa Cruz Biotechnology), phospho-T^389^-S6K (#9205, CST), total S6K (#9202, CST), phospho-T^346^-NDRG1 (#3217, CST), total NDRG1 (#ab124689, Abcam), phospho-S^473^-AKT (#9271, CST), total AKT (#9272, CST), Rictor (#A300-459A, Bethyl Laboratories), actin (#ab3280, Abcam), α-GST (#2622, CST), α-myc (#2276, CST), α-Flag (#F3165, Sigma), Ki-67 (#ab15580, Abcam).

**Yeast methods:** Standard techniques were used for yeast manipulations (1). Yeast reagents used are derivatives of the MATCHMAKER Gal4 Two-Hybrid System 3 (Clontech). The drug sensitive screening strain AR109D (*MAT****a****, trp1-901, leu2-3, 112, ura3-52, his3-200, gal4∆, gal80∆, LYS::GAL1_UAS_-GAL1_TATA_-HIS3, GAL2_UAS_-GAL2_TATA_-ADE2, URA3::MEL1_UAS_-MEL1_TATA_-lacZ, avo3::KanMX, tor2::KanMX pRS316::Ypk2^D239A^-HA, pdr1:CUP1pro-HXT9, pdr3:CUP1pro-HXT11)* was derived from AR109 (2). Expression of *Ypk2^D239A^* (constitutively active Ypk2 (D239A) allele) was well tolerated in this strain and conferred viability in the absence of *Avo3* and *Tor2* (3).

**CID613034 analog preparation:**

**Chemistry.** *General*

All reactions were carried out under open-air condition unless otherwise specified. Dichloromethane (DCM) and triethylamine (TEA) were distilled from calcium hydride under an argon atmosphere. Chemicals were purchased from Sigma-Aldrich, Alfa aesar and TCI in ≥95% purity, all other solvents or reagents were purified according to literature procedures if necessary. ^1^H-NMR spectra were recorded on Bruker spectrometers at 400 or 500 MHz and are reported relative to deuterated solvent signals (CHCl_3_ δ 7.26; DMSO δ 2.48 ppm). Data for ^1^H NMR spectra are reported as follows: chemical shift (δ ppm), multiplicity, coupling constant (Hz) and integration. Splitting patterns are designated as follows: s, singlet; d, doublet; dd, doublet of doublets; ddd, doublet of doublets of doubles; dt, doublet of triplets; t, triplet; td, triplet of doublets; q, quartet; sext, sextet; m, multiplet; and br, broad. ^13^C NMR spectra were recorded on Bruker Spectrometers at 125 MHz and are reported relative to deuterated solvent signals (CHCl_3_ δ 77.0; DMSO δ 40.0 ppm). Data for ^13^C spectra are reported in terms of chemical shift. The chemical shifts are reported in parts per million (ppm, δ). Melting points were obtained using Buchi B-545 melting point apparatus and are uncorrected. The reactions were monitored with a silica gel TLC plate under UV light (254 and 365 nm) followed by visualization with a ninhydrin or phosphomolybdic acid staining solution. Column chromatography was performed on silica gel 60, 230-400 mesh. DART-HRMS spectra were collected on a Thermo Exactive Plus MSD (Thermo Scientific) equipped with an ID-CUBE ion source and a Vapur Interface (IonSense). Both the source and MSD were controlled by Excalibur, version 3.0. The purity of the compounds was assayed by high field proton and carbon NMR and was ≥95%.

**Synthetic Procedures and Characterization Data**

Scheme 1. Synthesis of Compound CID613034 (JR-AB2-000)

**3,4-Dimethylphenylisothiocyanate, 1.** To a dichloromethane (100 mL) solution of 3,4-dimethylaniline (1212 mg, 10.0 mmol, 1.0 eq) was added thiophosgene (1.0 mL, 13.0 mmol, 1.3 eq) and triethylamine (3.49 mL, 25.0 mmol, 2.5 eq) at 0 ^o^C. The mixture was warmed to room temperature and stirred for 3 h. After the reaction was completed, the mixture was diluted with dichloromethane (200 mL) and washed with water (100 mL X 2). The combined organic layer was dehydrated with brine and MgSO_4_, and then concentrated in vacuo. The residue was purified by flash column chromatography over silica gel (hexane/ethyl acetate, 50:1, v/v) to afford the desired product **1** (1.548 g, 95%) as pale yellow oil: Rf = 0.6 (hexane/ethyl acetate, 50:1, v/v); ^1^H NMR (DMSO-d6, 500 MHz) δ 7.21 (s, 1H), 7.18 (d, *J* = 8.0 Hz, 1H), 7.13 (dd, *J* = 8.0, 2.0 Hz, 1H), 2.20 (s, 3H), 2.19 (s, 3H); ^13^C NMR (DMSO-d6, 125 MHz) δ 138.8, 137.3, 133.0, 131.1, 127.7, 127.1, 123.5, 19.6, 19.5.

**1-(3,4-Dimethylphenyl)-3-(2-hydroxypropyl)thiourea, 2.** (4) To an acetone (20 mL) solution of 3,4-dimethylphenylisothiocyanate (**1**, 460 mg, 2.87 mmol, 1.0 eq) was added *DL*-1-amino-2-propanol (0.29 mL, 3.73 mmol, 1.3 eq) dropwise at room temperature. The mixture was refluxed for 3 h, and then concentrated in vacuo. The residue was purified by flash column chromatography over silica gel (hexane/ethyl acetate, 1:1, v/v) to afford the desired product **2** (810 mg, 91%) as pale yellow oil: Rf = 0.25 (hexane/ethyl acetate, 1:1, v/v); ^1^H NMR (CDCl_3_, 500 MHz) δ 7.68 (br, 1H), 7.16 (d, *J* = 7.5 Hz, 1H), 6.97 (s, 1H), 6.95 (d, *J* = 8.0 Hz, 1H), 6.44 (br, 1H), 4.04-4.01 (m, 1H), 3.91-3.88 (m, 1H), 3.44 (ddd, *J* = 13.5, 5.0, 8.0 Hz, 1H), 2.25 (s, 6H), 1.19 (d, *J* = 6.5 Hz, 3H); ^13^C NMR (CDCl_3_, 125 MHz) δ 181.3, 138.9, 136.4, 133.4. 131.2, 126.5, 122.7, 67.3, 52.1, 21.2, 19.9, 19.4.

***N*-(3,4-Dimethylphenyl)-5-methyl-4,5-dihydrothiazol-2-amine, 3.** (4) To an ethanol (10 mL) solution of 1-(3,4-dimethylphenyl)-3-(2-hydroxypropyl)thiourea (**2**, 810 mg, 3.4 mmol, 1.0 eq) was added conc. HCl (10 mL) at room temperature, and the mixture was refluxed for 2 h. After the completion of reaction, the mixture was diluted with ethyl acetate (100 mL) and quenched with aq. NaHCO_3_ to make HCl salt to free form. The organic layer was dehydrated with brine and MgSO_4_ and then concentrated in vacuo. The residue was purified by flash column chromatography over silica gel (hexane/ethyl acetate, 1:1, v/v) to afford the desired product **3** (516 mg, 68%) as pale brown solid: Rf = 0.2 (hexane/ethyl acetate, 1:1, v/v); ^1^H NMR (CDCl_3_, 500 MHz) δ 7.03 (d, *J* = 8.0 Hz, 1H), 6.90 (s, 1H), 6.86 (d, *J* = 8.0 Hz, 1H), 3.89-3.84 (m, 2H), 3.45-3.44 (m, 1H), 2.22 (s, 3H), 2.20 (s, 3H), 1.42 (d, *J* = 6.5 Hz, 3H); ^13^C NMR (CDCl_3_, 125 MHz) δ 161.5, 144.6, 137.2, 131.7, 130.0, 122.5, 118.5, 57.8, 43.7, 20.4, 19.9, 19.2.

**3-(3,4-Dichlorophenyl)-1-(3,4-dimethylphenyl)-1-(5-methyl-4,5-dihydrothiazol-2-yl)urea, JR-AB2-000.** A toluene (10 mL) solution of *N*-(3,4-dimethylphenyl)-5-methyl-4,5-dihydrothiazol-2-amine (**3**, 510 mg, 2.3 mmol, 1.0 eq) and 3,4-dichlorophenyl isocyanate (433 mg, 2.3 mmol, 1.0 eq) was refluxed for 2 h under argon gas atmosphere. After the completion of reaction, the mixture was concentrated in vacuo, and the residue was crystallized using ethyl acetate and hexane to afford the desired product **JR-AB2-000** (690 mg, 73%) as white powder: Rf = 0.2 (hexane/ethyl acetate, 5:1, v/v); mp 158-159 ^o^C; ^1^H NMR (CDCl_3_, 500 MHz) δ 12.2 (s, 1H), 7.73 (s, 1H), 7.33 (d, *J* = 1.0 Hz, 2H), 7.13 (d, *J* = 8.0 Hz, 1H), 6.79 (d, *J* = 8.0 Hz, 1H), 6.74 (dd, *J* = 8.0, 2.0 Hz, 1H), 4.44 (dd, *J* = 11.5, 6.5 Hz, 1H), 3.95 (dd, *J* = 11.5, 6.5 Hz, 1H), 3.70 (sext, *J* = 6.5 Hz, 1H), 2.28 (s, 3H), 2.26 (s, 3H), 1.45 (d, *J* = 6.5 Hz, 3H); ^13^C NMR (CDCl_3_, 125 MHz) δ 158.3, 150.9, 146.6, 137.9, 137.7, 133.5, 132.7, 130.4, 130.3, 126.6, 122.7, 121.4, 119.1, 118.5, 55.5, 36.9, 20.0, 19.9, 19.3; DART-HRMS found 408.0689 [M+H]^+^, calcd for C_19_H_20_Cl_2_N_3_OS 408.0704.

Scheme 2. Synthesis of JR-AB2-001

***N*-(3,4-Dimethylphenyl)-4,5-dihydrothiazol-2-amine, 4.** (4) To an acetone (20 mL) solution of 3,4-dimethylphenylisothiocyanate (**1**, 326 mg, 2.0 mmol, 1.0 eq) was added aminoethanol (0.19 mL, 2.6 mmol, 1.3 eq) dropwise at room temperature. The mixture was refluxed for 2 h, and then concentrated in vacuo. The crude mixture was dissolved in ethanol (10 mL) and conc. HCl (10 mL) at room temperature, and the mixture was refluxed for 2 h. After the completion of reaction, the mixture was diluted with ethyl acetate (100 mL) and quenched with aq. NaHCO_3_ to make HCl salt to free form. The organic layer was dehydrated with brine and MgSO_4_ and then concentrated in vacuo. The residue was purified by flash column chromatography over silica gel (hexane/ethyl acetate, 1:1, v/v) to afford the desired product **4** (404 mg, 98%) as pale yellow solid: Rf = 0.15 (hexane/ethyl acetate, 1:1, v/v); ^1^H NMR (CDCl_3_, 500 MHz) δ 7.03 (d, *J* = 8.0 Hz, 1H), 6.94 (s, 1H), 6.91 (d, *J* = 7.5 Hz, 1H), 3.85 (t, *J* = 7.0 Hz, 2H), 3.30 (t, *J* = 7.0 Hz, 2H), 2.23 (s, 3H), 2.21 (s, 3H); ^13^C NMR (CDCl_3_, 125 MHz) δ 160.5, 144.2, 137.2, 131.5, 130.1, 122.2, 118.1, 32.4, 29.7, 19.9, 19.2.

**3-(3,4-Dichlorophenyl)-1-(4,5-dihydrothiazol-2-yl)-1-(3,4-dimethylphenyl)urea, JR-AB2-001.** A toluene (10 mL) solution of *N*-(3,4-dimethylphenyl)-4,5-dihydrothiazol-2-amine (**4**, 206 mg, 1.0 mmol, 1.0 eq) and 3,4-dichlorophenyl isocyanate (188 mg, 1.0 mmol, 1.0 eq) was refluxed for 1 h. After the completion of reaction, the mixture was concentrated in vacuo, and the residue was crystallized using ethyl acetate and hexane to afford the desired product **JR-AB2-001** (280 mg, 71%) as white powder: Rf = 0.5 (hexane/ethyl acetate, 3:1, v/v); mp 166-168 ^o^C; ^1^H NMR (CDCl_3_, 500 MHz) δ 12.2 (s, 1H), 7.73 (t, *J* = 1.0 Hz, 1H), 7.32 (d, *J* = 1.0 Hz, 2H), 7.13 ( d, *J* = 8.0 Hz, 1H), 6.80 (s, 1H), 6.75 (dd, *J* = 8.0, 2.0 Hz, 1H), 4.36 (t, *J* = 7.0 Hz, 2H), 3.18 (t, *J* = 7.0 Hz, 2H), 2.28 (s, 3H), 2.27 (s, 3H); ^13^C NMR (CDCl_3_, 125 MHz) δ 158.3, 150.8, 146.6, 137.8, 137.7, 133.5, 132.7, 130.39, 130.38, 126.6, 122.6, 121.4, 119.1, 118.4, 48.8, 25.7, 19.9, 19.3; DART-HRMS found 394.0560 [M+H]^+^, calcd for C_18_H_18_Cl_2_N_3_OS 394.0548.

Scheme 3. Synthesis of JR-AB2-002

**1-(3,4-Dimethylphenyl)-3-(2-hydroxy-2-methylpropyl)thiourea, 5.** (4,5) To a diethyl ether (30 mL) suspension of lithium aluminum hydride (380 mg, 10.0 mmol, 2.0 eq) was added acetone cyanohydrin (0.46 mL, 5.0 mmol, 1.0 eq) in diethyl ether (20 mL) dropwise at 0 ^o^C. The mixture was stirred for 2 h at room temperature. To the reaction mixture was added excess amount of sodium sulfate hydrate at 0 ^o^C with vigorous stirring, and the mixture was stirred for 4 h at room temperature. The resulting mixture was filtered through celite and the residue was washed with diethyl ether. The ether washings were all combined and dehydrated with MgSO4 and then concentrated in vacuo. The crude mixture was dissolved in acetone (20 mL) and added 3,4-dimethylphenyl isothiocyanate (**1**, 571 mg, 3.5 mmol, 0.7 eq), then the mixture was refluxed for 2 h and concentrated in vacuo. The residue was purified by flash column chromatography over silica gel (hexane/ethyl acetate, 10:1 🡪 3:1, v/v) to afford the desired product **5** (287 mg, 33% based on 1) as pale yellow solid: Rf = 0.15 (hexane/ethyl acetate, 3:1, v/v); ^1^H NMR (CDCl_3_, 500 MHz) δ 7.66 (br, 1H), 7.16 (d, *J* = 5.5 Hz, 1H), 6.98 (s, 1H), 6.97 (d, *J* = 6.0 Hz, 1H), 6.45 (s, 1H), 3.68 (s, 2H), 2.25 (s, 6H), 2.00 (br, 1H), 1.23 (s, 6H); ^13^C NMR (CDCl_3_, 125 MHz) δ 181.4, 138.9, 136.4, 133.4, 131.2, 126.5, 122.7, 71.4, 55.5, 27.6, 19.9, 19.4.

***N*-(3,4-Dimethylphenyl)-5,5-dimethyl-4,5-dihydrothiazol-2-amine, 6.** (4) To an acetone (10 mL) solution of 1-(3,4-dimethylphenyl)-3-(2-hydroxy-2-methylpropyl)thiourea (**5**, 252 mg, 1.0 mmol, 1.0 eq) was added conc. HCl (5 mL) and the mixture was refluxed for 24 h. After the completion of reaction, the mixture was diluted with ethyl acetate (100 mL) and quenched with aq. NaHCO_3_ to make HCl salt to free form. The organic layer was dehydrated with brine and MgSO_4_ and then concentrated in vacuo. The residue was purified by flash column chromatography over silica gel (hexane/ethyl acetate, 10:1 🡪 2:1, v/v) to afford the desired product **6** (53 mg, 23%) as white powder: Rf = 0.15 (hexane/ethyl acetate, 1:1, v/v); ^1^H NMR (CDCl_3_, 400 MHz) δ 7.03 (d, *J* = 8.0 Hz, 1H), 6.88 (s, 1H), 6.84 (d, *J* = 8.0 Hz, 1H), 3.51 (s, 2H), 2.22 (s, 3H), 2.20 (s, 3H), 1.54 (s, 6H).

**3-(3,4-Dichlorophenyl)-1-(5,5-dimethyl-4,5-dihydrothiazol-2-yl)-1-(3,4-dimethylphenyl)urea, JR-AB2-002.** A toluene (2 mL) solution of *N*-(3,4-dimethylphenyl)-5,5-dimethyl-4,5-dihydrothiazol-2-amine (**6**, 43 mg, 0.18 mmol, 1.0 eq) and 3,4-dichlorophenyl isocyanate (35 mg, 0018 mmol, 1.0 eq) was refluxed for 1 h. After the completion of reaction, the mixture was concentrated in vacuo, and the residue was crystallized using ethyl acetate and hexane to afford the desired product **JR-AB2-002** (57 mg, 75%) as white powder: Rf = 0.7 (hexane/ethyl acetate, 3:1, v/v); mp 161-162 ^o^C; ^1^H NMR (CDCl_3_, 500 MHz) δ 12.3 (s, 1H), 7.74 (t, *J* = 1.5 Hz, 1H), 7.33 (d, *J* = 1.5 Hz, 2H), 7.12 (d, *J* = 8.0 Hz, 1H), 6.78 (s, 1H), 6.73 (dd, *J* = 8.0, 2.0 Hz, 1H), 4.09 (s, 2H), 2.28 (s, 3H), 2.26 (s, 3H), 1.54 (s, 6H); ^13^C NMR (CDCl_3_, 125 MHz) δ 158.4, 151.1, 146.4, 137.9, 137.7, 133.5, 132.7, 130.4, 130.3, 126.5, 122.7, 121.4, 119.1, 118.5, 61.1, 47.5, 28.3, 20.0, 19.3; DART-HRMS found 422.0837 [M+H]^+^, calcd for C_20_H_22_Cl_2_N_3_OS 422.0861.

Scheme 4. Synthesis of JR-AB2-003

**4-Methoxyphenyl isothiocyanate, 7.** To a dichloromethane (100 mL) solution of p-anisidine (1232 mg, 10.0 mmol, 1.0 eq) was added thiophosgene (1.0 mL, 13.0 mmol, 1.3 eq) and triethylamine (3.49 mL, 25.0 mmol, 2.5 eq) at 0 ^o^C. The mixture was warmed to room temperature and stirred for 2 h. After the reaction was completed, the mixture was diluted with dichloromethane (200 mL) and washed with water (100 mL X 2). The combined organic layer was dehydrated with brine and MgSO_4_, and then concentrated in vacuo. The residue was purified by flash column chromatography over silica gel (hexane/ethyl acetate, 10:1, v/v) to afford the desired product **7** (1.274 g, 77%) as yellow oil: Rf = 0.8 (hexane/ethyl acetate, 5:1, v/v); ^1^H NMR (CDCl_3_, 500 MHz) δ 7.17 (ddd, *J* = 9.0, 3.0, 2.5 Hz, 2H), 6.85 (ddd, *J* = 9.5, 3.0, 2.5 Hz, 2H), 3.81 (s, 3H); ^13^C NMR (CDCl_3_, 125 MHz) δ 158.7, 134.0, 127.1, 123.7, 114.9, 55.7.

**1-(2-Hydroxypropyl)-3-(4-methoxyphenyl)thiourea, 8.** (4) To an acetone (20 mL) solution of 4-methoxyphenyl isothiocyanate (**7**, 374 mg, 2.26 mmol, 1.0 eq) was added *DL*-1-amino-2-propanol (0.23 mL, 2.94 mmol, 1.3 eq) dropwise at room temperature. The mixture was refluxed for 1 h, and then concentrated in vacuo. The residue was purified by flash column chromatography over silica gel (hexane/ethyl acetate, 5:1 🡪 1:1, v/v) to afford the desired product **8** (532 mg, 98%) as pale brown caramel: Rf = 0.1 (hexane/ethyl acetate, 3:1, v/v); ^1^H NMR (CDCl_3_, 500 MHz) δ 7.83 (br, 1H), 7.15 (d, *J* = 8.0 Hz, 2H), 6.92 (d, *J* = 8.5 Hz, 2H), 6.32 (br, 1H), 4.02-3.99 (m, 1H), 3.87-3.85 (m, 1H), 3.42 (ddd, *J* = 14.0, 5.0, 3.0 Hz, 1H), 2.31 (br, 1H), 1.18 (d, *J* = 6.5 Hz, 3H); ^13^C NMR (CDCl_3_, 125 MHz) δ 181.7, 159.1, 128.5, 127.7, 115.4, 67.3, 55.7, 52.1, 21.2.

***N*-(4-Methoxyphenyl)-5-methyl-4,5-dihydrothiazol-2-amine, 9.** (4) To an ethanol (10 mL) solution of 1-(2-hydroxypropyl)-3-(4-methoxyphenyl)thiourea (**8**, 580 mg, 2.41 mmol, 1.0 eq) was added conc. HCl (10 mL) at room temperature, and the mixture was refluxed for 1 h. After the completion of reaction, the mixture was diluted with ethyl acetate (100 mL) and quenched with aq. NaHCO_3_ to make HCl salt to free form. The organic layer was dehydrated with brine and MgSO_4_ and then concentrated in vacuo. The residue was purified by flash column chromatography over silica gel (hexane/ethyl acetate, 1:1, v/v) to afford the desired product 9 (457 mg, 85%) as pale yellow solid: Rf = 0.15 (hexane/ethyl acetate, 1:1, v/v); ^1^H NMR (CDCl_3_, 500 MHz) δ 7.02 (d, *J* = 9.0 Hz, 2H), 6.83 (d, *J* = 9.0 Hz, 2H), 3.88-3.80 (m, 2H), 3.78 (s, 3H), 3.39 (dd, *J* = 10.0, 5.5 Hz, 1H), 1.42 (d, *J* = 6.5 Hz, 3H); ^13^C NMR (CDCl_3_, 125 MHz) δ 161.6, 155.9, 141.5, 122.6, 114.3, 57.2, 55.6, 43.6, 20.5.

**3-(4-(Dimethylamino)phenyl)-1-(4-methoxyphenyl)-1-(5-methyl-4,5-dihydrothiazol-2-yl)urea, JR-AB2-003.** (6) To a dichloromethane solution (3 mL) of triphosgene (149 mg, 0.5 mmol, 1.0 eq) was added 4-*N*,*N*-dimethylamino-aniline (68 mg, 0.5 mmol, 1.0 eq) in dichloromethane (2 mL) and triethylamine (0.15 mL. 1.1 mmol, 2.2 eq) dropwise. After 30 min stirring at room temperature, dichloromethane was removed in vacuo. To the toluene (3 mL) solution of the crude mixture was added *N*-(4-methoxyphenyl)-5-methyl-4,5-dihydrothiazol-2-amine (**9**, 111 mg, 0.5 mmol, 1.0 eq) in toluene (2 mL) and the mixture was refluxed for 3 h. After the completion of reaction, the mixture was concentrated in vacuo, and the residue was diluted with ethyl acetate (50 mL) and washed with aq. NaHCO_3_ solution (20 mL). The organic layer was dehydrated with brine and MgSO_4_ and then concentrated in vacuo. The residue was purified by flash column chromatography over silica gel (hexane/ethyl acetate, 3:1, v/v) to afford the desired product **JR-AB2-003** (84 mg, 44% for 2 steps) as light yellow solid: Rf = 0.3 (hexane/ethyl acetate, 3:1, v/v); mp 143-145 ^o^C; ^1^H NMR (CDCl_3_, 500 MHz) δ 11.7 (s, 1H), 7.36 (d, *J* = 8.0 Hz, 2H), 6.94 (d, *J* = 7.5 Hz, 2H), 6.89 (d, *J* = 7.5 Hz, 2H), 6.71 (d, *J* = 8.0 Hz, 2H), 4.45 (dd, *J* = 11.5, 6.5 Hz, 1H), 3.96 (dd, *J* = 11.5, 6.5 Hz, 1H), 3.81 (s, 3H), 3.68 (sext, *J* = 6.5 Hz, 1H), 2.91 (s, 6H), 1.44 (d, *J* = 6.5 Hz, 3H); ^13^C NMR (CDCl_3_, 125 MHz) δ 158.0, 156.8, 151.3, 147.7, 142.7, 128.1, 122.5, 121.8, 114.3, 113.3, 55.7, 55.5, 41.1, 36.9, 20.0; DART-HRMS found 385.1697 [M+H]^+^, calcd for C_20_H_25_N_4_O_2_S 385.1698.

Scheme 5. Synthesis of JR-AB2-004

**3,4-Dichlorophenyl isothiocyanate, 10.** To a dichloromethane (20 mL) solution of 3,4-dichloroaniline (324 mg, 2.0 mmol, 1.0 eq) was added thiophosgene (0.2 mL, 2.6 mmol, 1.3 eq) and triethylamine (0.7 mL, 5.0 mmol, 2.5 eq) at 0 ^o^C. The mixture was warmed to room temperature and stirred for 30 min. After the reaction was completed, the mixture was diluted with ethyl acetate (100 mL) and washed with water (50 mL X 2). The combined organic layer was dehydrated with brine and MgSO_4_, and then concentrated in vacuo. The residue was purified by flash column chromatography over silica gel (hexane/ethyl acetate, 50:1, v/v) to afford the desired product **10** (323 mg, 79%) as pale yellow oil: Rf = 0.85 (hexane/ethyl acetate, 5:1, v/v).

**3-(3,4-Dichlorophenyl)-1-(3,4-dimethylphenyl)-1-(5-methyl-4,5-dihydrothiazol-2-yl)thiourea, JR-AB2-004.** A toluene solution of *N*-(3,4-dimethylphenyl)-5-methyl-4,5-dihydrothiazol-2-amine (**3**, 220 mg, 1.0 mmol, 1.0 eq) and phenyl 3,4-dichlorophenyl isothiocyanate (**10**, 204 mg, 1.0 mmol, 1.0 eq) was refluxed for 2 h. After the completion of reaction, the mixture was concentrated in vacuo, and the residue was purified by flash column chromatography over silica gel (hexane/ethyl acetate, 20:1, v/v) to afford the desired product **JR-AB2-004** (72 mg, 72%) as white powder: Rf = 0.4 (hexane/ethyl acetate, 10:1, v/v); mp 124-126 ^o^C; ^1^H NMR (CDCl_3_, 500 MHz) δ 14.3 (s, 1H), 7.79 (d, *J* = 2.0 Hz, 1H), 7.45 (dd, *J* = 9.0, 3.0 Hz, 1H), 7.40 (d, *J* = 9.0 Hz, 1H), 7.13 (d, *J* = 9.0 Hz, 1H), 6.78 (s, 1H), 6.73 (dd, *J* = 7.5, 2.0 Hz, 1H), 5.01 (dd, *J* = 12.0, 6.5 Hz, 1H), 4.39 (dd, *J* = 12.0, 6.5 Hz, 1H), 3.69 (sext, *J* = 6.5 Hz, 1H), 2.27 (s, 3H), 2.26 (s, 3H), 1.46 (d, *J* = 7.0 Hz, 3H); ^13^C NMR (CDCl_3_, 125 MHz) δ 179.0, 159.1, 145.7, 138.3, 137.8, 134.0, 132.4, 130.4, 130.2, 129.6, 126.3, 124.1, 122.5, 118.3, 61.5, 35.8, 19.9, 19.7, 19.3; DART-HRMS found 424.0258 [M+H]^+^, calcd for C_19_H_20_Cl_2_N_3_S_2_ 424.0476.

Scheme 6. Synthesis of JR-AB2-005

**3-(3,4-Dichlorophenyl)-1-(4-methoxyphenyl)-1-(5-methyl-4,5-dihydrothiazol-2-yl)urea, JR-AB2-005.** A toluene (5 mL) solution of *N*-(4-methoxyphenyl)-5-methyl-4,5-dihydrothiazol-2-amine (**9**, 67 mg, 0.3 mmol, 1.0 eq) and 3,4-dichlorophenyl isocyanate (56.4 mg, 0.3 mmol, 1.0 eq) was refluxed for 1 h. After the completion of reaction, the mixture was concentrated in vacuo, and the residue was the residue was purified by flash column chromatography over silica gel (hexane/ethyl acetate, 5:1, v/v) to afford the desired product **JR-AB2-005** (114 mg, 93%) as white powder: Rf = 0.5 (hexane/ethyl acetate, 2:1, v/v); mp 129-131 ^o^C; ^1^H NMR (CDCl_3_, 500 MHz) δ 12.3 (s, 1H), 7.75 (s, 1H), 7.33 (s, 2H), 6.95 (d, *J* = 8.0 Hz, 2H), 6.91 (d, *J* = 8.0 Hz, 2H), 4.44 (dd, *J* = 11.0, 7.0 Hz, 1H), 3.95 (dd, *J* = 11.0, 7.0 Hz, 1H), 3.82 (s, 3H), 3.71 (sext, *J* = 6.0 Hz, 1H), 1.46 (d, *J* = 6.0 Hz, 3H); ^13^C NMR (CDCl_3_, 125 MHz) δ 158.5, 157.1, 150.9, 142.0, 137.9, 132.7, 130.4, 126.6, 122.5, 121.4, 119.2, 114.5, 55.54, 55.53, 36.9, 20.0; DART-HRMS found 410.0464 [M+H]^+^, calcd for C_18_H_18_Cl_2_N_3_O_2_S 410.0497.

Scheme 7. Synthesis of JR-AB2-006

***N*-(3,4-Dimethylphenyl)-5-methyl-4,5-dihydrooxazol-2-amine, 11.** (4) To a methanol (10 mL) solution of 1-(3,4-dimethylphenyl)-3-(2-hydroxypropyl)thiourea (**2**, 238 mg, 1.0 mmol, 1.0 eq) was iodomethane (0.31 mL, 5.0 mmol, 5.0 eq), and the mixture was stirred for 4 h. After the completion of reaction, the mixture was concentrated in vacuo to remove the excess reagent and solvent. To the crude mixture was added 2.5M KOH in methanol (10 mL, 25.0 mmol, 25.0 eq) and the mixture was stirred for 2 h at room temperature. After the completion of reaction, the mixture was diluted with ethyl acetate (100 mL) and washed with water (30 mL X2). The organic layer was dehydrated with brine and MgSO_4_ and then concentrated in vacuo. The desired product **11** (192 mg, 94%) was adducted as white solid without further purification: Rf = 0.4 (dichloromethane/methanol, 10:1, v/v); ^1^H NMR (CDCl_3_, 500 MHz) δ 7.06 (s, 1H), 7.02 (s, 2H), 4.73 (sext, *J* = 6.0 Hzm 1H), 4.40 (br, 1H), 3.91 (dd, *J* = 11.0, 8.0 Hz, 1H), 3.39 (dd, *J* = 11.0, 7.5 Hz, 1H), 2.22 (s, 3H), 2.19 (s, 3H), 1.41 (d, *J* = 6.0 Hz, 3H).

**3-(3,4-Dichlorophenyl)-1-(3,4-dimethylphenyl)-1-(5-methyl-4,5-dihydrooxazol-2-yl)urea, JR-AB2-006.** A toluene (10 mL) solution of *N*-(3,4-dimethylphenyl)-5-methyl-4,5-dihydrooxazol-2-amine (**11**, 102 mg, 0.5 mmol, 1.0 eq) and 3,4-dichlorophenyl isocyanate (94 mg, 0.5 mmol, 1.0 eq) was refluxed for 20 min. After the completion of reaction, the mixture was concentrated in vacuo, and the residue was crystallized using ethyl acetate and hexane to afford the desired product **JR-AB2-006** (192 mg, 98%) as white powder: Rf = 0.4 (hexane/ethyl acetate, 3:1, v/v); mp 183-184 ^o^C; ^1^H NMR (CDCl_3_, 500 MHz) δ 12.0 (s, 1H), 7.77 (s, 1H), 7.35 (s, 2H), 7.09 (d, *J* = 8.0 Hz, 1H), 6.93 (s, 1H), 6.90 (dd, *J* = 8.0, 2.0 Hz, 1H), 4.79 (sext, *J* = 6.5 Hz, 1H), 4.25 (dd, *J* = 10.5, 8.0 Hz, 1H), 3.67 (dd, *J* = 10.5, 7.5 Hz, 1H), 2.27 (s, 3H), 2.24 (s, 3H), 1.51 (d, *J* = 6.5 Hz, 3H); ^13^C NMR (CDCl_3_, 125 MHz) δ 150.0, 149.4, 141.6, 137.8, 137.1, 132.7, 132.5, 130.5, 130.0, 126.5, 124.5, 121.1, 120.4, 118.8, 73.5, 49.4, 20.1, 19.9, 19.2; DART-HRMS found 392.0919 [M+H]^+^, calcd for C_19_H_20_Cl_2_N_3_O_2_ 392.0933.

Scheme 8. Synthesis of Compound JR-AB2-007

**1-(2-Hydroxypropyl)-3-phenylthiourea, 12.** (4) To an acetone (20 mL) solution of phenyl isothiocyanate (0.597 mL, 5.0 mmol, 1.0 eq) was added *DL*-1-amino-2-propanol (0.502 mL, 6.5 mmol, 1.3 eq) dropwise at room temperature. The mixture was refluxed for 1 h, and then concentrated in vacuo. The crude mixture was directly used for the synthesis of compound 13: Rf = 0.2 (hexane/ethyl acetate, 1:1, v/v).

**5-Methyl-*N*-phenyl-4,5-dihydrothiazol-2-amine, 13.** (4) To an ethanol (10 mL) solution of 1-(2-hydroxypropyl)-3-phenylthiourea (**12**, 5.0 mmol, 1.0 eq) was added conc. HCl (5 mL) at room temperature, and the mixture was refluxed for 2 h. After the completion of reaction, the mixture was diluted with ethyl acetate (100 mL) and quenched with aq. NaHCO_3_ to make HCl salt to free form. The organic layer was dehydrated with brine and MgSO_4_ and then concentrated in vacuo. The residue was purified by flash column chromatography over silica gel (hexane/ethyl acetate, 1:1 🡪 0:1, v/v) to afford the desired product **13** (881 mg, 92% for 2 steps) as white solid: Rf = 0.15 (hexane/ethyl acetate, 1:1, v/v).

**3-(3,4-Dichlorophenyl)-1-(5-methyl-4,5-dihydrothiazol-2-yl)-1-phenylurea, JR-AB2-007.** A toluene (20 mL) solution of 5-methyl-*N*-phenyl-4,5-dihydrothiazol-2-amine (**13**, 385 mg, 2.0 mmol, 1.0 eq) and 3,4-dichlorophenyl isocyanate (376 mg, 2.0 mmol, 1.0 eq) was refluxed for 2 h. After the completion of reaction, the mixture was concentrated in vacuo, and the residue was purified by flash column chromatography over silica gel (hexane/ethyl acetate, 1:0 🡪 10:1, v/v) to afford the desired product **JR-AB2-007** (720 mg, 95%) as white powder: Rf = 0.8 (hexane/ethyl acetate, 1:1, v/v); mp 126-128 ^o^C; ^1^H NMR (CDCl_3_, 500 MHz) δ 12.2 (s, 1H), 7.74 (s, 1H), 7.38 (t, *J* = 7.5 Hz, 2H), 7.33 (s, 2H), 7.19 (t, *J* = 7.5 Hz, 1H), 7.00 (d, *J* = 7.5 Hz, 2H), 4.45 (dd, *J* = 11.5, 7.0 Hz, 1H), 3.97 (dd, *J* = 11.5, 7.0 Hz, 1H), 3.72 (sext, *J* = 6.5 Hz, 1H), 1.46 (d, *J* = 6.5 Hz, 3H); ^13^C NMR (CDCl_3_, 125 MHz) δ 158.8, 150.8, 148.8, 137.8, 132.7, 130.4, 129.3, 126.7, 125.1, 121.50, 121.48, 119.2, 55.6, 37.0, 20.0; DART-HRMS found 380.0361 [M+H]^+^, calcd for C_17_H_16_Cl_2_N_3_OS 380.0391.

Scheme 9. Synthesis of Compound JR-AB2-008

**1-(4-(Dimethylamino)phenyl)-3-(2-hydroxypropyl)thiourea, 14.** (4) To an acetone (20 mL) solution of 4-Dimethylaminophenyl isothiocyanate (535 mg, 3.0 mmol, 1.0 eq) was added *DL*-1-amino-2-propanol (0.301 mL, 3.9 mmol, 1.3 eq) dropwise at room temperature. The mixture was refluxed for 1 h, and then concentrated in vacuo. The crude mixture was directly used for the synthesis of compound 15: Rf = 0.2 (hexane/ethyl acetate, 1:1, v/v).

***N_1_*,*N_1_*-Dimethyl-*N_4_*-(5-methyl-4,5-dihydrothiazol-2-yl)benzene-1,4-diamine, 15.** (4) To an ethanol (10 mL) solution of 1-(4-(dimethylamino)phenyl)-3-(2-hydroxypropyl)thiourea (**14**, 3.0 mmol, 1.0 eq) was added conc. HCl (5 mL) at room temperature, and the mixture was refluxed for 2 h. After the completion of reaction, the mixture was diluted with ethyl acetate (100 mL) and quenched with aq. NaHCO_3_ to make HCl salt to free form. The organic layer was dehydrated with brine and MgSO_4_ and then concentrated in vacuo. The residue was purified by flash column chromatography over silica gel (hexane/ethyl acetate, 1:1 🡪 ethyl acetate/methanol, 10:1, v/v) to afford the desired product **15** (600 mg, 85% for 2 steps) as brown solid: Rf = 0.2 (hexane/ethyl acetate, 1:1, v/v); ^1^H NMR (CDCl_3_, 500 MHz) δ 7.03 (d, *J* = 8.0 Hz, 2H), 6.69 (d, *J* = 8.0 Hz, 2H), 3.90-3.86 (m, 2H), 3.47-3.45 (m, 1H), 2.91 (s, 6H), 1.43 (d, *J* = 6.0 Hz, 3H); ^13^C NMR (CDCl_3_, 125 MHz) δ 163.2, 147.9, 135.6, 123.0, 113.3, 47.9, 43.9, 41.0, 20.5.

**3-(3,4-Dichlorophenyl)-1-(4-(dimethylamino)phenyl)-1-(5-methyl-4,5-dihydrothiazol-2-yl)urea, JR-AB2-008.** A toluene (20 mL) solution of *N_1_*,*N_1_*-dimethyl-*N_4_*-(5-methyl-4,5-dihydrothiazol-2-yl)benzene-1,4-diamine (**15**, 471 mg, 2.0 mmol, 1.0 eq) and 3,4-dichlorophenyl isocyanate (376 mg, 2.0 mmol, 1.0 eq) was refluxed for 2 h. After the completion of reaction, the mixture was concentrated in vacuo, and the residue was purified by flash column chromatography over silica gel (hexane/ethyl acetate, 1:0 🡪 10:1, v/v) to afford the desired product **JR-AB2-008** (761 mg, 90%) as white powder: Rf = 0.6 (hexane/ethyl acetate, 1:1, v/v); mp 135-137 ^o^C; ^1^H NMR (CDCl_3_, 500 MHz) δ 12.4 (s, 1H), 7.75 (s, 1H), 7.33 (s, 2H), 6.93 (dd, *J* = 9.0, 2.0 Hz, 2H), 6.75 (dd, *J* = 9.0, 2.0 Hz, 2H), 4.42 (ddd, *J* = 11.0, 6.5, 2.0 Hz, 1H), 4.94 (ddd, *J* = 11.0, 6.5, 2.0 Hz, 1H), 3.69 (sext, *J* = 6.5 Hz, 1H), 2.96 (s, 6H), 1.45 (d, *J* = 6.5 Hz, 3H); ^13^C NMR (CDCl_3_, 125 MHz) δ 157.4, 151.1, 148.4, 138.5, 138.0, 132.6, 130.4, 126.4, 122.2, 121.4, 119.1, 113.1, 55.4, 40.9, 36.8, 20.0; DART-HRMS found 423.0786 [M+H]^+^, calcd for C_19_H_21_Cl_2_N_4_OS 423.0813.

Scheme 10. Synthesis of Compound JR-AB2-009

**1-(2-Hydroxypropyl)-3-(*p*-tolyl)thiourea, 16.** (4) To an acetone (20 mL) solution of 4-Methylphenyl isothiocyanate (448 mg, 3.0 mmol, 1.0 eq) was added *DL*-1-amino-2-propanol (0.301 mL, 3.9 mmol, 1.3 eq) dropwise at room temperature. The mixture was refluxed for 2 h, and then concentrated in vacuo. The crude mixture was directly used for the synthesis of compound 17: Rf = 0.2 (hexane/ethyl acetate, 1:1, v/v).

**5-Methyl-N-(*p*-tolyl)-4,5-dihydrothiazol-2-amine, 17.** (4) To an ethanol (10 mL) solution of 1-(2-hydroxypropyl)-3-(*p*-tolyl)thiourea (**16**, 3.0 mmol, 1.0 eq) was added conc. HCl (5 mL) at room temperature, and the mixture was refluxed for 3 h. After the completion of reaction, the mixture was diluted with ethyl acetate (100 mL) and quenched with aq. NaHCO_3_ to make HCl salt to free form. The organic layer was dehydrated with brine and MgSO_4_ and then concentrated in vacuo. The residue was purified by flash column chromatography over silica gel (hexane/ethyl acetate, 1:1, v/v) to afford the desired product **17** (600 mg, 97% for 2 steps) as brown solid: Rf = 0.2 (hexane/ethyl acetate, 1:1, v/v); ^1^H NMR (CDCl_3_, 500 MHz) δ 7.08 (d, *J* = 8.0 Hz, 2H), 6.98 (d, *J* = 8.0 Hz, 2H), 5.10 (br, 1H), 3.87-3.80 (m, 2H), 3.39 (dd, *J* = 10.0, 6.0 Hz, 1H), 2.30 (s, 3H), 1.42 (d, *J* = 6.5 Hz, 3H); ^13^C NMR (CDCl_3_, 125 MHz) δ 161.5, 145.5, 132.6, 129.5, 121.2, 57.0, 43.4, 20.9, 20.4.

**3-(3,4-Dichlorophenyl)-1-(5-methyl-4,5-dihydrothiazol-2-yl)-1-(*p*-tolyl)urea, JR-AB2-009.** A toluene (10 mL) solution of 5-methyl-N-(*p*-tolyl)-4,5-dihydrothiazol-2-amine (**17**, 206 mg, 1.0 mmol, 1.0 eq) and 3,4-dichlorophenyl isocyanate (188 mg, 1.0 mmol, 1.0 eq) was refluxed for 2 h. After the completion of reaction, the mixture was concentrated in vacuo, and the residue was crystallized using ethyl acetate to afford the desired product **JR-AB2-009** (236 mg, 60%) as white powder: Rf = 0.6 (hexane/ethyl acetate, 3:1, v/v); mp 160-162 ^o^C; ^1^H NMR (CDCl_3_, 500 MHz) δ 12.2 (s, 1H), 7.74 (s, 1H), 7.33 (s, 2H), 7.18 (d, *J* = 8.0 Hz, 2H), 6.90 (d, *J* = 8.5 Hz, 2H), 4.44 (dd, *J* = 11.5, 6.5 Hz, 1H), 3.96 (dd, *J* = 11.5, 6.5 Hz, 1H), 3.71 (sext, *J* = 6.5 Hz, 1H), 2.36 (s, 3H), 1.45 (d, *J* = 7.0 Hz, 3H); ^13^C NMR (CDCl_3_, 125 MHz) δ 158.5, 150.9, 146.2, 137.8, 134.8, 132.7, 130.4, 129.9, 126.6, 121.4, 121.3, 119.1, 55.6, 36.9, 21.0, 20.0; DART-HRMS found 394.0531 [M+H]^+^, calcd for C_18_H_18_Cl_2_N_3_OS 394.0548.

Scheme 11. Synthesis of Compound JR-AB2-010

**1-(2-Hydroxypropyl)-3-(*m*-tolyl)thiourea, 18.** (4) To an acetone (20 mL) solution of 3-methylphenyl isothiocyanate (448 mg, 3.0 mmol, 1.0 eq) was added *DL*-1-amino-2-propanol (0.301 mL, 3.9 mmol, 1.3 eq) dropwise at room temperature. The mixture was refluxed for 2 h, and then concentrated in vacuo. The residue was purified by flash column chromatography over silica gel (hexane/ethyl acetate, 3:1 🡪 1:1, v/v) to afford the desired product **18** (646 mg, 96%) as pale yellow solid: Rf = 0.3 (hexane/ethyl acetate, 1:1, v/v); ^1^H NMR (CDCl_3_, 500 MHz) δ 7.73 (br, 1H), 7.30 (t, *J* = 8.0 Hz, 1H), 7.10 (d, *J* = 7.5 Hz, 1H), 7.02 (s, 2H), 6.51 (br, 1H), 4.08-4.00 (m, 1H), 3.98-3.85 (m, 1H), 3.45 (ddd, *J* = 13.5, 7.5, 5.0 Hz, 1H), 2.36 (s, 3H), 1.21 (d, *J* = 6.5 Hz, 3H); ^13^C NMR (CDCl_3_, 125 MHz) δ 181.1, 140.5, 135.9, 130.0, 128.2, 125.7, 122.1, 67.2, 52.1, 21.4, 21.2.

**5-Methyl-*N*-(*m*-tolyl)-4,5-dihydrothiazol-2-amine, 19.** (4) To an ethanol (10 mL) solution of 1-(2-hydroxypropyl)-3-(*m*-tolyl)thiourea (**18**, 630mg, 2.81 mmol, 1.0 eq) was added conc. HCl (5 mL) at room temperature, and the mixture was refluxed for 3 h. After the completion of reaction, the mixture was diluted with ethyl acetate (100 mL) and quenched with aq. NaHCO_3_ to make HCl salt to free form. The organic layer was dehydrated with brine and MgSO_4_ and then concentrated in vacuo. The residue was purified by flash column chromatography over silica gel (hexane/ethyl acetate, 1:1, v/v) to afford the desired product **19** (538 mg, 93%) as brown solid: Rf = 0.3 (hexane/ethyl acetate, 1:1, v/v); ^1^H NMR (CDCl_3_, 500 MHz) δ 7.16 (t, *J* = 7.0 Hz, 1H), 6.93 (s, 1H), 6.91 (d, *J* = 6.5 Hz, 1H), 6.85 (d, *J* = 6.5 Hz, 1H), 3.89-3.82 (m, 2H), 3.44-3.41 (m, 1H), 2.31 (s, 3H), 1.42 (d, *J* = 6.5 Hz, 3H); ^13^C NMR (CDCl_3_, 125 MHz) δ 161.1, 147.5, 138.8, 128.8, 124.0, 121.8, 118.1, 57.6, 43.7, 21.5, 20.4.

**3-(3,4-Dichlorophenyl)-1-(5-methyl-4,5-dihydrothiazol-2-yl)-1-(*m*-tolyl)urea, JR-AB2-010.** A toluene (10 mL) solution of 5-methyl-*N*-(*m*-tolyl)-4,5-dihydrothiazol-2-amine (**19**, 206 mg, 1.0 mmol, 1.0 eq) and 3,4-dichlorophenyl isocyanate (188 mg, 1.0 mmol, 1.0 eq) was refluxed for 2 h. After the completion of reaction, the mixture was concentrated in vacuo, and the residue was purified by flash column chromatography over silica gel (hexane/ethyl acetate, 10:1, v/v) to afford the desired product **JR-AB2-010** (273 mg, 69%) as white powder: Rf = 0.7 (hexane/ethyl acetate, 3:1, v/v); mp 111-113 ^o^C; ^1^H NMR (CDCl_3_, 500 MHz) δ 12.2 (s, 1H), 7.74 (s, 1H), 7.33 (s, 2H), 7.26 (t, *J* = 7.5 Hz, 1H), 7.00 (d, *J* = 7.5 Hz, 1H), 6.82 (s, 1H), 6.80 (d, *J* = 8.0 Hz, 1H), 4.45 (dd, *J* = 11.5, 6.5 Hz, 1H), 3.96 (dd, *J* = 11.5, 6.5 Hz, 1H), 3.71 (sext, *J* = 6.5 Hz, 1H), 2.38 (s, 3H), 1.46 (d, *J* = 7.0 Hz, 3H); ^13^C NMR (CDCl_3_, 125 MHz) δ 158.5, 150.8, 148.7, 139.3, 137.8, 132.7, 130.4, 129.1, 126.6, 125.9, 122.2, 121.5, 119.2, 118.3, 55.6, 36.9, 21.5, 20.0; DART-HRMS found 394.0526 [M+H]^+^, calcd for C_18_H_18_Cl_2_N_3_OS 394.0548.

Scheme 12. Synthesis of Compound JR-AB2-011

**1-(4-Fluorophenyl)-3-(2-hydroxypropyl)thiourea, 20.** (4) To an acetone (20 mL) solution of 4-fluorophenyl isothiocyanate (460 mg, 3.0 mmol, 1.0 eq) was added *DL*-1-amino-2-propanol (0.301 mL, 3.9 mmol, 1.3 eq) dropwise at room temperature. The mixture was refluxed for 2 h, and then concentrated in vacuo. The residue was purified by flash column chromatography over silica gel (hexane/ethyl acetate, 3:1 🡪 1:1, v/v) to afford the desired product **20** (566 mg, 83%) as pale yellow solid: Rf = 0.3 (hexane/ethyl acetate, 1:1, v/v); ^1^H NMR (CDCl_3_, 500 MHz) δ 7.67 (br, 1H), 7.25-7.22 (m, 2H), 7.12 (t, *J* = 8.0 Hz, 2H), 6.34 (br, 1H), 4.08-4.00 (m, 1H), 3.99-3.82 (m, 1H), 3.42 (ddd, *J* = 13.5, 8.0, 5.0 Hz, 1H), 1.95 (br, 1H), 1.21 (d, *J* = 6.5 Hz, 3H); ^13^C NMR (CDCl_3_, 125 MHz) δ 181.6. 161.5 (d, *J* = 248.8 Hz, 1C), 131.9, 127.8, 117.2 (d, *J* = 21 Hz, 2C), 67.1, 52.1, 21.2.

***N*-(4-Fluorophenyl)-5-methyl-4,5-dihydrothiazol-2-amine, 21.** (4) To an ethanol (10 mL) solution of 1-(4-fluorophenyl)-3-(2-hydroxypropyl)thiourea (**20**, 550 mg, 2.41 mmol, 1.0 eq) was added conc. HCl (5 mL) at room temperature, and the mixture was refluxed for 3 h. After the completion of reaction, the mixture was diluted with ethyl acetate (100 mL) and quenched with aq. NaHCO_3_ to make HCl salt to free form. The organic layer was dehydrated with brine and MgSO_4_ and then concentrated in vacuo. The residue was purified by flash column chromatography over silica gel (hexane/ethyl acetate, 1:1, v/v) to afford the desired product **21** (472 mg, 93%) as pale brown solid: Rf = 0.3 (hexane/ethyl acetate, 1:1, v/v); ^1^H NMR (CDCl_3_, 500 MHz) δ 7.04-6.99 (m, 2H), 6.96 (t, J = 9.0 Hz, 2H), 3.86 (sext, *J* = 6.5 Hz, 1H), 3.78 (dd, *J* = 10.0, 6.5 Hz, 1H), 3.36 (dd, *J* = 10.0. 6.5 Hz, 1H), 1.43 (d, *J* = 6.5 Hz, 3H); ^13^C NMR (CDCl_3_, 125 MHz) δ 162.0, 159.1 (d, *J* = 240.0 Hz, 1C), 145.0, 122.7 (d, *J* = 7.5 Hz, 2C), 115.5 (d, *J* = 22.5 Hz, 2C), 55.8, 43.2, 20.3.

**3-(3,4-Dichlorophenyl)-1-(4-fluorophenyl)-1-(5-methyl-4,5-dihydrothiazol-2-yl)urea, JR-AB2-011.** A toluene (10 mL) solution of *N*-(4-fluorophenyl)-5-methyl-4,5-dihydrothiazol-2-amine (**21**, 210 mg, 1.0 mmol, 1.0 eq) and 3,4-dichlorophenyl isocyanate (188 mg, 1.0 mmol, 1.0 eq) was refluxed for 3 h. After the completion of reaction, the mixture was concentrated in vacuo, and the residue was purified by flash column chromatography over silica gel (hexane/ethyl acetate, 10:1, v/v) to afford the desired product **JR-AB2-011** (272 mg, 68%) as white powder: Rf = 0.7 (hexane/ethyl acetate, 3:1, v/v); mp 126-128 ^o^C; ^1^H NMR (CDCl_3_, 500 MHz) δ 12.1 (s, 1H), 7.73 (d, *J* = 2.0 Hz, 1H), 7.34 (d, *J* = 8.5 Hz, 1H), 7.32 (dd, *J* = 8.5, 2.0 Hz, 1H), 7.06 (td, *J* = 8.5, 2.0 Hz, 2H), 6.95 (ddd, *J* = 9.0, 5.0, 2.0, 2H), 4.45 (dd, *J* = 11.5, 7.0 Hz, 1H), 3.97 (dd, *J* = 11.5, 6.5 Hz, 1H)m 3.72 (sext, *J* = 6.5 Hz, 1H), 1.46 (d, *J* = 7.0 Hz, 3H); ^13^C NMR (CDCl_3_, 125 MHz) δ 159.4, 158.7 (d, *J* = 243.8 Hz, 1C), 150.7, 144.9, 137.7, 132.7, 130.4, 126.8, 122.9 (d, *J* = 8.8 Hz, 2C), 121.5, 119.2, 116.0 (d, *J* = 22.5 Hz, 2C), 55.7, 37.0, 20.0; DART-HRMS found 398.0266 [M+H]^+^, calcd for C_17_H_14_Cl_2_FN_3_OS 398.0297.

Scheme 13. Synthesis of Compound JR-AB2-012

**1-Ethyl-3-(2-hydroxypropyl)thiourea, 22.** (4) To an acetone (20 mL) solution of ethyl isothiocyanate (0.26 mL, 3.0 mmol, 1.0 eq) was added *DL*-1-amino-2-propanol (0.301 mL, 3.9 mmol, 1.3 eq) dropwise at room temperature. The mixture was refluxed for 2 h, and then concentrated in vacuo. The residue was purified by flash column chromatography over silica gel (hexane/ethyl acetate, 2:1 🡪 1:1, v/v) to afford the desired product **22** (274 mg, 56%) as white powder: Rf = 0.15 (hexane/ethyl acetate, 1:1, v/v).

***N*-Ethyl-5-methyl-4,5-dihydrothiazol-2-amine, 23.** (4) To an ethanol (10 mL) solution of 1-ethyl-3-(2-hydroxypropyl)thiourea (**22**, 250mg, 1.54 mmol, 1.0 eq) was added conc. HCl (5 mL) at room temperature, and the mixture was refluxed for 3 h. After the completion of reaction, the mixture was diluted with ethyl acetate (100 mL) and quenched with aq. NaHCO_3_ to make HCl salt to free form. The organic layer was dehydrated with brine and MgSO_4_ and then concentrated in vacuo. The desired product **23** (130 mg, 59%) was afforded without further purification as yellow solid: Rf = 0.05 (hexane/ethyl acetate, 0:1, v/v); ^1^H NMR (CDCl_3_, 500 MHz) δ 4.01 (dd, *J* = 13.0, 7.0 Hz, 1H), 3.92 (sext, *J* = 7.0 Hz, 1H), 3.65 (dd, *J* = 13.0, 5.0 Hz, 1H), 3.30 (q, *J* = 7.5 Hz, 2H), 1.37 (d, *J* = 6.5 Hz, 3H), 1.18 (t, *J* = 7.5 Hz, 3H); ^13^C NMR (CDCl_3_, 125 MHz) δ 160.8, 67.5, 47.8, 39.6, 21.3, 15.1.

**3-(3,4-Dichlorophenyl)-1-ethyl-1-(5-methyl-4,5-dihydrothiazol-2-yl)urea, JR-AB2-012.** A toluene (10 mL) solution of *N*-ethyl-5-methyl-4,5-dihydrothiazol-2-amine (**23**, 101 mg, 0.7 mmol, 1.0 eq) and 3,4-dichlorophenyl isocyanate (132 mg, 0.7 mmol, 1.0 eq) was refluxed for 3 h. After the completion of reaction, the mixture was concentrated in vacuo, and the residue was purified by flash column chromatography over silica gel (hexane/ethyl acetate, 10:1, v/v) to afford the desired product **JR-AB2-012** (218 mg, 94%) as white powder: Rf = 0.7 (hexane/ethyl acetate, 3:1, v/v); mp 114-115 ^o^C; ^1^H NMR (CDCl_3_, 500 MHz) δ 12.6 (s, 1H), 7.71 (d, *J* = 2.5 Hz, 1H), 7.33 (d, *J* = 9.0 Hz, 1H), 7.28 (dd, *J* = 9.0, 2.5 Hz, 1H), 4.32 (dd, *J* = 11.5, 6.5 Hz, 1H), 3.85 (dd, *J* = 11.5, 6.5 Hz, 1H), 3.72 (sext, *J* = 6.5 Hz, 1H), 3.29 (d, *J* = 7.5 Hz, 2H), 1.47 (d, *J* = 7.0 Hz, 3H), 1.31 (t, *J* = 7.5 Hz, 3H); ^13^C NMR (CDCl_3_, 125 MHz) δ 156.4, 151.4, 138.3, 132.6, 130.4, 126.2, 121.2, 118.9, 54.9, 49.4, 37.1, 20.3, 16.1; DART-HRMS found 332.0367 [M+H]^+^, calcd for C_13_H_16_Cl_2_N_3_OS 332.0391.

Scheme 14. Synthesis of Compound JR-AB2-013

***N*-(3,4-Dimethylphenyl)thiazol-2-amine, 24.** (7) To an isopropanol (25 mL) solution of 3,4-dimethylaniline (606mg, 5.0 mmol, 1.0 eq) and p-toluenesulfonic acid hydrate (476 mg, 2.5 mmol, 0.5 eq) was added 2-bromothiazole (0.68 mL. 7.5 mmol, 1.5 eq) at room temperature, and the mixture was refluxed for 120 h. After the completion of reaction, the mixture was diluted with ethyl acetate (100 mL) and quenched with aq. NaHCO_3_. The organic layer was dehydrated with brine and MgSO_4_ and then concentrated in vacuo. The residue was purified by flash column chromatography over silica gel (hexane/ethyl acetate, 10:1 🡪 5:1, v/v) to afford the desired product **24** (738 mg, 72%) as pale yellow solid: Rf = 0.2 (hexane/ethyl acetate, 5:1, v/v); ^1^H NMR (CDCl_3_, 500 MHz) δ 7.26 (d, *J* = 3.5 Hz, 1H), 7.12-7.09 (m, 3H), 6.58 (d, *J* = 3.5 Hz, 1H), 2.27 (s, 3H), 2.24 (s, 3H); ^13^C NMR (CDCl_3_, 125 MHz) δ 166.6, 138.7, 138.4, 137.9, 131.7, 130.5, 120.2, 116.1, 107.1, 20.0, 19.1.

**3-(3,4-Dichlorophenyl)-1-(3,4-dimethylphenyl)-1-(thiazol-2-yl)urea, JR-AB2-013.** A toluene (20 mL) solution of *N*-(3,4-dimethylphenyl)thiazol-2-amine (**24**, 480 mg, 2.35 mmol, 1.0 eq) and 3,4-dichlorophenyl isocyanate (442 mg, 2.35 mmol, 1.0 eq) was refluxed for 1 h. After the completion of reaction, the mixture was concentrated in vacuo, and the residue was purified by flash column chromatography over silica gel (hexane/ethyl acetate, 10:1, v/v) and the resulting white caramel was crystallized using dichloromethane and hexane to afford the desired product **JR-AB2-013** (663 mg, 72%) as white powder: Rf = 0.6 (hexane/ethyl acetate, 5:1, v/v); mp 122-123 ^o^C; ^1^H NMR (CDCl_3_, 500 MHz) δ 11.5 (br, 1H), 7.83 (d, *J* = 2.0 Hz, 1H), 7.42 (d, *J* = 3.5 Hz, 1H), 7.40 (d, *J* = 2.0 Hz, 1H), 7.35 (d, *J* = 8.5 Hz, 1H), 7.32 (d, *J* = 8.0 Hz, 1H), 7.16 (s, 1H), 7.13 (d, *J* = 8.0 Hz, 1H), 6.84 (d, *J* = 3.5 Hz, 1H), 2.34 (s, 3H), 2.31 (s, 3H); ^13^C NMR (CDCl_3_, 125 MHz) δ 167.4, 151.3, 139.1, 138.8, 138.3, 138.0, 137.4, 132.7, 131.5, 130.4, 130.0, 126.6, 126.4, 121.5, 119.1, 113.1, 20.0, 19.7; DART-HRMS found 392.0376 [M+H]^+^, calcd for C_18_H_16_Cl_2_N_3_OS 392.0391.

Scheme 15. Synthesis of Compound JR-AB2-014

***N*-(3,4-Dimethylphenyl)benzo[*d*]thiazol-2-amine, 25.** (8) To a dimethyl sulfoxide (4 mL) solution of 2-iodoaniline (438 mg, 2.0 mmol, 1.0 eq), 3,4-dimethylphenyl isothiocyanate (359 mg, 2.2 mmol, 1.1 eq), copper(II) sulfate (3.2 mg, 0.02 mmol, 0.01 eq) and tetrabutylamminium bromide (7.9 mg, 2.2 mmol, 1.1 eq) was stirred for 3 h at 80 ^o^C. After the completion of reaction, the mixture was diluted with ethyl acetate (100 mL) and washed with water. The organic layer was dehydrated with brine and MgSO_4_ and then concentrated in vacuo. The residue was purified by flash column chromatography over silica gel (hexane/ethyl acetate, 10:1, v/v) to afford the desired product **25** (479 mg, 94%) as yellow solid: Rf = 0.2 (hexane/ethyl acetate, 5:1, v/v); ^1^H NMR (CDCl_3_, 500 MHz) δ 7.61 (dd, *J* = 8.0, 0.5 Hz, 1H), 7.59 (d, *J* = 8.0 Hz, 1H), 7.32 (td, *J* = 8.0, 1.0 Hz, 1H), 7.23-7.20 (m, 2H), 7.15 (d, *J* = 8.5 Hz, 1H), 7.14 (td, *J* = 8.0, 1.0 Hz, 1H), 2.29 (s, 3H), 2.27 (s, 3H); ^13^C NMR (CDCl_3_, 125 MHz) δ 165.0, 151.6, 138.0, 137.4, 133.2, 130.6, 130.1, 126.1, 122.3, 122.1, 120.8, 119.4, 118.1, 20.0, 19.3.

**1-(Benzo[*d*]thiazol-2-yl)-3-(3,4-dichlorophenyl)-1-(3,4-dimethylphenyl)urea, JR-AB2-014.** A toluene (10 mL) solution of *N*-(3,4-dimethylphenyl)benzo[*d*]thiazol-2-amine (**25**, 254 mg, 1.0 mmol, 1.0 eq) and 3,4-dichlorophenyl isocyanate (188 mg, 1.0 mmol, 1.0 eq) was refluxed for 2 h. After the completion of reaction, the mixture was concentrated in vacuo, and the residue was purified by flash column chromatography over silica gel (hexane/ethyl acetate, 10:1, v/v) to afford the desired product **JR-AB2-014** (375 mg, 85%) as white powder: Rf = 0.8 (hexane/ethyl acetate, 5:1, v/v); mp 175-177 ^o^C; ^1^H NMR (CDCl_3_, 500 MHz) δ 12.3 (s, 1H), 7.91 (d, *J* = 2.5 Hz, 1H), 7.82 (d, *J* = 8.0 Hz, 1H), 7.59 (d, *J* = 8.0 Hz, 1H), 7.47 (td, *J* = 9.0, 2.5 Hz, 1H), 7.44 (dd, *J* = 9.0, 1.0 Hz,1H), 7.40 (d, *J* = 9.0 Hz, 1H), 7.33 (d, *J* = 8.0 Hz, 1H), 7.26 (td, *J* = 7.5, 1.0 Hz, 1H), 7.20 (d, *J* = 1.5 Hz, 1H), 7.16 (dd, *J* = 8.0, 1.5 Hz,1H), 2.36 (s, 3H), 2.33 (s, 3H); ^13^C NMR (CDCl_3_, 125 MHz) δ 166.5, 151.1, 150.1, 139.04 138.99, 138.0, 137.2, 132.8, 131.5, 131.3, 130.5, 130.2, 126.8, 126.62, 126.59, 124.0, 121.6, 120.8, 120.7, 119.2, 20.0, 19.7; DART-HRMS found 442.0516 [M+H]^+^, calcd for C_22_H_18_Cl_2_N_3_OS 442.0548.

Scheme 16. Synthesis of Compound JR-AB2-015

**1-(3,4-Dimethylphenyl)-3-(1-hydroxy-2-methylpropan-2-yl)thiourea, 26.** (4) To an acetone (20 mL) solution of 3,4-dimethylphenyl isothiocyanate (490 mg, 3.0 mmol, 1.0 eq) was added 2-amino-2-methyl-1-propanol (0.372 mL, 3.9 mmol, 1.3 eq) dropwise at room temperature. The mixture was refluxed for 1 h, and then concentrated in vacuo. The residue was crystallized using ethyl acetate and hexane to afford the desired product **26** (720 mg, 95%) as white powder: Rf = 0.4 (hexane/ethyl acetate, 1:1, v/v); ^1^H NMR (CDCl_3_, 500 MHz) δ 7.91 (br, 1H), 7.15 (d, *J* = 8.0 Hz, 1H), 6.98 (s, 1H), 6.97 (d, *J* = 8.0 Hz, 1H), 6.11 (br, 1H), 3.83 (s, 2H), 3.17 (br, 1H), 2.25 (s, 6H), 1.39 (s, 6H); ^13^C NMR (CDCl_3_, 125 MHz) δ 179.6, 138.6, 135.8, 134.3, 130.9, 126.1, 122.3, 69.3, 57.7, 24.8, 19.9, 19.4.

***N*-(3,4-Dimethylphenyl)-4,4-dimethyl-4,5-dihydrothiazol-2-amine, 27.** (4) To an ethanol (10 mL) solution of 1-(3,4-dimethylphenyl)-3-(1-hydroxy-2-methylpropan-2-yl)thiourea (**26**, 600mg, 2.52 mmol, 1.0 eq) was added conc. HCl (5 mL) at room temperature, and the mixture was refluxed for 1 h. After the completion of reaction, the mixture was diluted with ethyl acetate (100 mL) and quenched with aq. NaHCO_3_ to make HCl salt to free form. The organic layer was dehydrated with brine and MgSO_4_ and then concentrated in vacuo. The desired product **27** (545 mg, 92%) was afforded without further purification as white powder: Rf = 0.3 (hexane/ethyl acetate, 2:1, v/v); ^1^H NMR (CDCl_3_, 500 MHz) δ 7.09 (d, *J* = 8.0 Hz, 1H), 6.96 (d, *J* = 2.0 Hz, 1H), 6.93 (dd, *J* = 8.0, 2.0 Hz, 1H), 3.17 (s, 2H), 2.233 (s, 3H), 2.228 (s, 3H), 1.51 (s, 6H); ^13^C NMR (CDCl_3_, 125 MHz) δ 165.6, 139.4, 137.8, 134.5, 130.3, 123.9, 119.8, 64.5, 42.9, 27.4, 19.8, 19.3.

**3-(3,4-Dichlorophenyl)-1-(4,4-dimethyl-4,5-dihydrothiazol-2-yl)-1-(3,4-dimethylphenyl)urea, JR-AB2-015.** A toluene (10 mL) solution of *N*-(3,4-dimethylphenyl)-4,4-dimethyl-4,5-dihydrothiazol-2-amine (**27**, 234 mg, 1.0 mmol, 1.0 eq) and 3,4-dichlorophenyl isocyanate (188 mg, 1.0 mmol, 1.0 eq) was refluxed for 18 h. After the completion of reaction, the mixture was concentrated in vacuo, and the residue was purified by flash column chromatography over silica gel (hexane/ethyl acetate, 10:1, v/v) to afford the desired product **JR-AB2-015** (140 mg, 33%) as white powder: Rf = 0.8 (hexane/ethyl acetate, 2:1, v/v); mp 143-144 ^o^C; ^1^H NMR (CDCl_3_, 500 MHz) δ 12.6 (s, 1H), 7.73 (d, *J* = 2.5 Hz, 1H), 7.30 (d, *J* = 9.0 Hz, 1H), 7.27 (dd, *J* = 9.0, 2.5 Hz, 1H), 7.13 (d, *J* = 8.0 Hz, 1H), 6.79 (s, 1H), 6.74 (dd, *J* = 7.5, 2.0 Hz, 1H), 2.98 (s, 2H), 2.28 (s, 3H), 2.27 (s, 3H), 1.78 (s, 6H); ^13^C NMR (CDCl_3_, 125 MHz) δ 160.2, 151.1, 146.4, 138.1, 137.7, 133.4, 132.6, 130.34, 130.26, 126.4, 122.8, 121.7, 119.4, 118.6, 67.4, 40.3, 26.3, 19.9, 19.3; DART-HRMS found 422.0841 [M+H]^+^, calcd for C_20_H_22_Cl_2_N_3_OS 422.0861.

Scheme 17. Synthesis of Compound JR-AB2-016

***N*-(3,4-Dimethylphenyl)pyridin-2-amine, 28.** (9) A mixture of 2-bromopyridine (4.77 mL, 50.0 mmol, 10.0 eq) and 3,4-dimethylaniline (606 mg, 5.0 mmol, 1.0 eq) was refluxed for 3 h at 160 ^o^C. After the completion of reaction, the mixture was cooled down to room temperature, and directly purified by flash column chromatography over silica gel (hexane/ethyl acetate, 10:1, v/v) to afford the desired product **28** (920 mg, 93%) as white powder: Rf = 0.15 (hexane/ethyl acetate, 5:1, v/v); ^1^H NMR (CDCl_3_, 500 MHz) δ 8.17 (dd, *J* = 5.0, 1.0 Hz, 1H), 7.45 (ddd, *J* = 9.0, 7.5, 1.5 Hz, 1H), 7.07 (q, *J* = 8.0 Hz, 2H), 7.04 (dd, *J* = 7.0, 2.5 Hz, 1H), 6.82 (d, *J* = 8.5 Hz, 1H), 6.68 (ddd, *J* = 7.0, 5.0, 1.0 Hz, 1H), 6.48 (br, 1H), 2.25 (s, 3H), 2.24 (s, 3H); ^13^C NMR (CDCl_3_, 125 MHz) δ 156.6, 148.5, 138.0, 137.62, 137.59, 131.6, 130.3, 122.7, 118.7, 114.6, 107.6, 20.0, 19.1.

**3-(3,4-Dichlorophenyl)-1-(3,4-dimethylphenyl)-1-(pyridin-2-yl)urea, JR-AB2-016.** A toluene (20 mL) solution of *N*-(3,4-dimethylphenyl)pyridin-2-amine (**28**, 595 mg, 3.0 mmol, 1.0 eq) and 3,4-dichlorophenyl isocyanate (564 mg, 3.0 mmol, 1.0 eq) was refluxed for 3 h. After the completion of reaction, the mixture was concentrated in vacuo, and the residue was crystallized using ethyl acetate and hexane to afford the desired product **JR-AB2-016** (950 mg, 82%) as white powder: Rf = 0.5 (hexane/ethyl acetate, 5:1, v/v); mp 133-134 ^o^C; ^1^H NMR (CDCl_3_, 500 MHz) δ 13.2 (s, 1H), 8.35 (ddd, *J* = 5.0, 2.0, 0.5, 1H), 7.90 (d, *J* = 2.5 Hz, 1H), 7.50 (ddd, *J* = 9.0, 7.5, 2.0 Hz, 1H), 7.44 (dd, *J* = 9.0, 2.5 Hz, 1H), 7.34 (d, *J* = 8.5 Hz, 1H), 7.29 (d, *J* = 8.0 Hz, 1H), 7.06 (d, *J* = 1.5 Hz, 1H), 7.03 (dd, *J* = 7.5, 2.5 Hz, 1H), 6.98 (ddd, *J* = 8.0, 5.0, 0.5 Hz, 1H), 6.42 (d, *J* = 8.5 Hz, 1H), 2.32 (s, 3H), 2.30 (s, 3H); ^13^C NMR (CDCl_3_, 125 MHz) δ 156.1, 153.5, 145.2, 138.81, 138.76, 138.5, 137.2, 137.1, 132.5, 131.3, 130.5, 130.2, 126.8, 125.9, 121.5, 119.2, 117.5, 114.0, 20.0, 19.6; DART-HRMS found 386.0801 [M+H]^+^, calcd for C_20_H_18_Cl_2_N_3_O 386.0827.

Scheme 18. Synthesis of Compound JR-AB2-017

**3,4-Dimethyl-N-phenylaniline, 29.** (10) An ethyl acetate (25 mL) suspension of 3,4-dimethylaniline (606 mg, 5.0 mmol, 1.0 eq), phenylboronic acid (1829 mg, 15.0 mmol, 3.0 eq), copper(II) acetate (182 mg, 1.0 mmol, 0.2 eq), potassium carbonate (676 mg, 5.0 mmol, 1.0 eq) and benzoic acid (305 mg, 2.5 mmol, 0.5 eq) was refluxed for 24 h, and then the mixture was concentrated in vacuo. The residue was purified by flash column chromatography over silica gel (hexane/ethyl acetate, 100:1, v/v) to afford the desired product **29** (270 mg, 27%) as yellow solid: Rf = 0.7 (hexane/ethyl acetate, 5:1, v/v); ^1^H NMR (CDCl_3_, 500 MHz) δ 7.23 (td, *J* = 7.5, 1.5 Hz, 2H), 7.04 (d, *J* = 8.0 Hz, 1H), 7.01 (dd, *J* = 7.5, 1.0 Hz, 1H), 6.90-6.85 (d, 3H), 5.57 (br, 1H), 2.23 (s, 3H), 2.21 (s, 3H); ^13^C NMR (CDCl_3_, 125 MHz) δ 144.0, 140.6, 137.6, 130.3, 129.7, 129.3, 120.3, 120.2, 116.9, 116.3, 20.0, 19.0.

**3-(3,4-dichlorophenyl)-1-(3,4-dimethylphenyl)-1-phenylurea, JR-AB2-017.** A toluene (10 mL) solution of 3,4-dimethyl-N-phenylaniline (**29**, 197 mg, 1.0 mmol, 1.0 eq) and 3,4-dichlorophenyl isocyanate (188 mg, 1.0 mmol, 1.0 eq) was refluxed for 1 h. After the completion of reaction, the mixture was concentrated in vacuo, and the residue was purified by flash column chromatography over silica gel (hexane/ethyl acetate, 10:1, v/v) and the following product was crystallized using ethyl acetate and hexane to afford the desired product **JR-AB2-017** (305 mg, 79%) as white powder: Rf = 0.4 (hexane/ethyl acetate, 10:1, v/v); mp 118-119 ^o^C; ^1^H NMR (CDCl_3_, 500 MHz) δ 7.56 (d, *J* = 2.5 Hz, 1H), 7.38-7.22 (m, 6H), 7.19-7.16 (m, 2H), 7.11 (s, 1H), 7.07 (d, *J* = 8.0 Hz, 1H), 6.47 (br, 1H), 2.28 (s, 3H), 2.26 (s, 3H); ^13^C NMR (CDCl_3_, 125 MHz) δ 153.2, 142.2, 139.2, 138.7, 138.2, 136.5, 132.6, 131.1, 130.3, 129.4, 129.0, 126.9, 126.4, 126.1, 125.3, 120.8, 118.5, 19.9, 19.4; DART-HRMS found 385.0857 [M+H]^+^, calcd for C_21_H_19_Cl_2_N_2_O 385.0874.

Scheme 19. Synthesis of JR-AB2-018

**1-(3,4-Dimethylphenyl)-1-(5-methyl-4,5-dihydrothiazol-2-yl)-3-phenylurea, JR-AB2-018.** A toluene solution of *N*-(3,4-dimethylphenyl)-5-methyl-4,5-dihydrothiazol-2-amine (**3**, 220 mg, 1.0 mmol, 1.0 eq) and phenyl isocyanate (0.109 mL, 1.0 mmol, 1.0 eq) was refluxed for 1 h under argon gas atmosphere. After the completion of reaction, the mixture was concentrated in vacuo, and the residue was crystallized using ethyl acetate and hexane to afford the desired product **JR-AB2-018** (280 mg, 88%) as white powder: Rf = 0.7 (hexane/ethyl acetate, 2:1, v/v); mp 142-143 ^o^C; ^1^H NMR (CDCl_3_, 500 MHz) δ 12.1 (s, 1H), 7.51 (d, *J* = 8.5 Hz, 2H), 7.30 (t, *J* = 7.5 Hz, 2H), 7.12 (d, *J* = 8.0 Hz, 1H), 7.06 (t, *J* = 7.5 Hz, 1H), 6.81 (s, 1H), 6.75 (dd, *J* = 8.0, 2.0 Hz, 1H), 4.46 (dd, *J* = 11.5, 6.5 Hz, 1H), 3.97 (dd, *J* = 11.5, 7.0 Hz, 1H), 3.69 (sext, *J* = 6.5 Hz, 1H), 2.28 (s, 3H), 2.26 (s, 3H), 1.45 (d, *J* = 7.0 Hz, 3H); ^13^C NMR (CDCl_3_, 125 MHz) δ 157.9, 151.1, 146.9, 138.2, 137.6, 133.2, 130.3, 128.9, 123.6, 122.7, 120.0, 118.5, 55.6, 36.8, 20.0, 19.9, 19.3; DART-HRMS found 340.1475 [M+H]^+^, calcd for C_19_H_22_N_3_OS 340.1484.

Scheme 20. Synthesis of JR-AB2-019

**3-(3-Chlorophenyl)-1-(3,4-dimethylphenyl)-1-(5-methyl-4,5-dihydrothiazol-2-yl)urea, JR-AB2-019.** (11) To a dichloromethane solution (5 mL) of triphosgene (110 mg, 0.37 mmol, 0.37 eq) was added 3-chloroaniline (0.106 mL, 1.0 mmol, 1.0 eq) in dichloromethane (3 mL) and triethylamine (0.14 mL. 1.1 mmol, 1.1 eq) dropwise. After 30 min stirring at room temperature, then *N*-(3,4-dimethylphenyl)-5-methyl-4,5-dihydrothiazol-2-amine (**3**, 220 mg, 1.0 mmol, 1.0 eq) in dichloromethane (3 mL) was added and the mixture was stirred 30 min at room temperature. After the completion of reaction, the mixture was concentrated in vacuo, and the residue was diluted with ethyl acetate (100 mL) and washed with aq. NaHCO_3_ solution (30 mL). The organic layer was dehydrated with brine and MgSO_4_ and then concentrated in vacuo. The residue was purified by flash column chromatography over silica gel (hexane/ethyl acetate, 5:1, v/v) to afford the desired product **JR-AB2-019** (200 mg, 53%) as white powder: Rf = 0.7 (hexane/ethyl acetate, 2:1, v/v); mp 148-149 ^o^C; ^1^H NMR (CDCl_3_, 500 MHz) δ 12.2 (s, 1H), 7.62 (t, *J* = 2.0 Hz, 1H), 7.35 (ddd, *J* = 8.0, 2.0, 1.0 Hz, 1H), 7.20 (t, *J* = 8.0 Hz, 1H), 7.13 (d, *J* = 8.0 Hz, 1H), 7.03 (ddd, *J* = 8.0, 2.0, 1.0 Jz, 1H), 6.80 (d, *J* = 2.0 Hz, 1H), 6.75 (dd, *J* = 8.0, 2.5 Hz, 1H), 4.45 (dd, *J* = 11.5, 6.5 Hz, 1H), 3.96 (dd, *J* = 11.5, 6.5 Hz, 1H), 3.70 (sext, *J* = 6.5 Hz, 1H), 2.28 (s, 3H), 2.26 (s, 3H), 1.45 (d, *J* = 6.5 Hz, 3H); ^13^C NMR (CDCl_3_, 125 MHz) δ 158.1, 151.0, 146.7, 139.5, 137.7, 134.6, 133.4, 130.3, 129.9, 123.6, 122.7, 119.9, 118.5, 117.9, 55.6, 36.9, 20.0, 19.9, 19.3; DART-HRMS found 374.1068 [M+H]^+^, calcd for C_19_H_21_ClN_3_OS 374.1094.

Scheme 21. Synthesis of JR-AB2-020

**3-(4-Chlorophenyl)-1-(3,4-dimethylphenyl)-1-(5-methyl-4,5-dihydrothiazol-2-yl)urea, JR-AB2-020.** A toluene solution of *N*-(3,4-dimethylphenyl)-5-methyl-4,5-dihydrothiazol-2-amine (**3**, 220 mg, 1.0 mmol, 1.0 eq) and 4-chlorophenyl isocyanate (153 mg, 1.0 mmol, 1.0 eq) was refluxed for 2 h under argon gas atmosphere. After the completion of reaction, the mixture was concentrated in vacuo, and the residue was crystallized using ethyl acetate and hexane to afford the desired product **JR-AB2-020** (310 mg, 83%) as white powder: Rf = 0.8 (hexane/ethyl acetate, 2:1, v/v); mp 140-142 ^o^C; ^1^H NMR (CDCl_3_, 500 MHz) δ 12.1 (s, 1H), 7.46 (dt, *J* = 9.0, 2.0 Hz, 2H), 7.26-7.24 (m, 2H), 7.12 (d, *J* = 8.0 Hz, 1H), 6.79 (d, *J* = 2.0 Hz, 1H), 6.74 (dd, *J* = 8.0, 2.0 Hz, 1H), 4.44 (dd, *J* = 11.5, 6.5 Hz, 1H), 3.96 (dd, *J* = 11.5, 7.0 Hz, 1H), 3.69 (sext, *J* = 6.5 Hz, 1H), 2.28 (s, 3H), 2.26 (s, 3H), 1.45 (d, *J* = 7.0 Hz, 3H); ^13^C NMR (CDCl_3_, 125 MHz) δ 158.1, 151.0, 146.7, 137.6, 136.9, 133.3, 130.3, 128.9, 128.5, 122.7, 121.1, 118.5, 55.6, 36.9, 20.0, 19.9, 19.3; DART-HRMS found 374.1081 [M+H]^+^, calcd for C_19_H_21_ClN_3_OS 374.1094.

Scheme 22. Synthesis of JR-AB2-021

**1-(3,4-Dimethylphenyl)-3-(4-fluorophenyl)-1-(5-methyl-4,5-dihydrothiazol-2-yl)urea, JR-AB2-021.** A toluene solution of *N*-(3,4-dimethylphenyl)-5-methyl-4,5-dihydrothiazol-2-amine (**3**, 220 mg, 1.0 mmol, 1.0 eq) and 4-fluorophenyl isocyanate (0.114 mL, 1.0 mmol, 1.0 eq) was refluxed for 1 h under argon gas atmosphere. After the completion of reaction, the mixture was concentrated in vacuo, and the residue was crystallized using ethyl acetate and hexane to afford the desired product **JR-AB2-021** (300 mg, 84%) as white powder: Rf = 0.7 (hexane/ethyl acetate, 2:1, v/v); mp 135-136 ^o^C; ^1^H NMR (CDCl_3_, 500 MHz) δ 12.0 (s, 1H), 7.47-7.45 (m, 2H), 7.12 (d, *J* = 7.5 Hz, 1H), 7.01-6.97 (m, 2H), 6.79 (d, *J* = 2.0 Hz, 1H), 6.74 (dd, *J* = 7.5, 2.0 Hz, 1H), 4.45 (dd, *J* = 11.5, 6.5, 1H), 3.96 (dd, *J* = 11.5, 7.0 Hz, 1H), 3.69 (sext, *J* = 6.5 Hz, 1H), 2.27 (s, 3H), 2.26 (s, 3H), 1.45 (d, *J* = 6.5 Hz, 3H); ^13^C NMR (CDCl_3_, 125 MHz) δ 159.1 (d, *J* = 241.3 Hz, 1C), 158.1, 151.2, 146.8, 137.6, 134.2 (d, *J* = 2.5 Hz, 1C), 133.3, 130.3, 122.7, 121.6 (d, *J* = 7.5 Hz, 2C), 118.5, 115.5 (d, *J* = 22.5 Hz, 2C), 55.6, 36.9, 20.0, 19.9, 19.3; DART-HRMS found 358.1378 [M+H]^+^, calcd for C_19_H_21_FN_3_OS 358.1389.

Scheme 23. Synthesis of JR-AB2-022

**1-(3,4-Dimethylphenyl)-1-(5-methyl-4,5-dihydrothiazol-2-yl)-3-(*p*-tolyl)urea, JR-AB2-022.** (11) To a dichloromethane solution (5 mL) of triphosgene (110 mg, 0.37 mmol, 0.37 eq) was added p-toluidine (107 mg, 1.0 mmol, 1.0 eq) in dichloromethane (3 mL) and triethylamine (0.14 mL. 1.1 mmol, 1.1 eq) dropwise. After 30 min stirring at room temperature, then *N*-(3,4-dimethylphenyl)-5-methyl-4,5-dihydrothiazol-2-amine (**3**, 220 mg, 1.0 mmol, 1.0 eq) in dichloromethane (3 mL) was added and the mixture was stirred 30 min at room temperature. After the completion of reaction, the mixture was concentrated in vacuo, and the residue was diluted with ethyl acetate (100 mL) and washed with aq. NaHCO_3_ solution (30 mL). The organic layer was dehydrated with brine and MgSO_4_ and then concentrated in vacuo. The residue was purified by flash column chromatography over silica gel (hexane/ethyl acetate, 5:1, v/v) to afford the desired product **JR-AB2-022** (260 mg, 74%) as white powder: Rf = 0.75 (hexane/ethyl acetate, 2:1, v/v); mp 129-130 ^o^C; ^1^H NMR (CDCl_3_, 500 MHz) δ 11.9 (s, 1H), 7.38 (dt, *J* = 7.5, 2.0 Hz, 2H), 7.12 (d, *J* = 7.5 Hz, 1H), 7.10 (d, *J* = 9.0 Hz, 2H), 6.80 (d, *J* = 2.0 Hz, 1H), 6.76 (dd, *J* = 7.5, 2.0 Hz, 1H), 4.45 (dd, *J* = 11.5, 6.5 Hz, 1H), 3.96 (dd, *J* = 11.5, 6.5 Hz, 1H), 3.68 (sext, *J* = 6.5 Hz, 1H), 2.30 (s, 3H), 2.27 (s, 3H), 2.26 (s, 3H), 1.44 (d, *J* = 6.5 Hz, 3H); ^13^C NMR (CDCl_3_, 125 MHz) δ 157.9, 151.1, 147.0, 137.6, 135.6, 133.1, 130.3, 129.4, 123.0, 122.7, 120.0, 118.5, 55.6, 36.8, 20.9, 20.0, 19.9, 19.3; DART-HRMS found 354.1617 [M+H]^+^, calcd for C_20_H_24_N_3_OS 354.1640.

Scheme 24. Synthesis of JR-AB2-023

**1-(3,4-Dimethylphenyl)-3-(4-methoxyphenyl)-1-(5-methyl-4,5-dihydrothiazol-2-yl)urea, JR-AB2-023.** (11) To a dichloromethane solution (5 mL) of triphosgene (110 mg, 0.37 mmol, 0.37 eq) was added *p*-anisidine (123 mg, 1.0 mmol, 1.0 eq) in dichloromethane (3 mL) and triethylamine (0.14 mL. 1.1 mmol, 1.1 eq) dropwise. After 30 min stirring at room temperature, then *N*-(3,4-dimethylphenyl)-5-methyl-4,5-dihydrothiazol-2-amine (**3**, 220 mg, 1.0 mmol, 1.0 eq) in dichloromethane (3 mL) was added and the mixture was stirred 30 min at room temperature. After the completion of reaction, the mixture was concentrated in vacuo, and the residue was diluted with ethyl acetate (100 mL) and washed with aq. NaHCO_3_ solution (30 mL). The organic layer was dehydrated with brine and MgSO_4_ and then concentrated in vacuo. The residue was purified by flash column chromatography over silica gel (hexane/ethyl acetate, 5:1, v/v) to afford the desired product **JR-AB2-023** (275 mg, 75%) as white powder: Rf = 0.6 (hexane/ethyl acetate, 2:1, v/v); mp 120-122 ^o^C; ^1^H NMR (CDCl_3_, 500 MHz) δ 11.9 (s, 1H), 7.41 (dt, *J* = 9.0, 2.0 Hz, 2H), 7.11 (d, *J* = 8.0 Hz, 1H), 6.84 (dt, *J* = 9.0, 2.0 Hz, 2H), 6.79 (d, *J* = 2.0 Hz, 1H), 6.74 (dd, *J* = 8.0, 2.0 Hz, 1H), 4.45 (dd, *J* = 11.5, 6.5 Hz, 1H), 3.96 (dd, *J* = 11.5, 6.5 Hz, 1H), 3.78 (s, 3H), 3.68 (sext, *J* = 6.5 Hz, 1H), 2.27 (s, 3H), 2.25 (s, 3H), 1.44 (d, *J* = 6.5 Hz, 3H); ^13^C NMR (CDCl_3_, 125 MHz) δ 157.9, 156.0, 151.3, 147.0, 137.6, 133.1, 131.3, 150.3, 122.7, 121.7, 118.5, 114.1, 55.6, 55.5, 36.9, 20.0 19.9, 19.3; DART-HRMS found 370.1565 [M+H]^+^, calcd for C_20_H_24_N_3_O_2_S 370.1589.

Scheme 25. Synthesis of JR-AB2-024

**1-(3,4-Dimethylphenyl)-1-(5-methyl-4,5-dihydrothiazol-2-yl)-3-(naphthalen-1-yl)urea, JR-AB2-024.** A toluene solution of *N*-(3,4-dimethylphenyl)-5-methyl-4,5-dihydrothiazol-2-amine (**3**, 220 mg, 1.0 mmol, 1.0 eq) and 1-naphthyl isocyanate (0.144 mL, 1.0 mmol, 1.0 eq) was refluxed for 2 h under argon gas atmosphere. After the completion of reaction, the mixture was concentrated in vacuo, and the residue was crystallized using ethyl acetate and hexane to afford the desired product **JR-AB2-024** (350 mg, 90%) as white powder: Rf = 0.6 (hexane/ethyl acetate, 2:1, v/v); mp 137-139 ^o^C; ^1^H NMR (CDCl_3_, 500 MHz) δ 12.6 (s, 1H), 8.24 (d, *J* = 8.0 Hz, 1H), 8.07 (d, *J* = 8.5 Hz, 1H), 7.83 (d, *J* = 8.0 Hz, 1H), 7.61 (d, *J* = 8.5 Hz, 1H), 7.48 (t, *J* = 8.0 Hz, 1H), 7.46-7.40 (m, 2H), 7.15 (d, *J* = 8.0 Hz, 1H), 6.91 (s, 1H), 6.85 (dd, *J* = 8.0, 2.0 Hz, 1H), 4.55 (ddd, *J* = 11.5, 6.5, 2.0 Hz, 1H), 4.04 (ddd, *J* = 11.5, 6.5, 2.0 Hz, 1H), 3.75 (sext, *J* = 6.5 Hz, 1H), 2.30 (s, 3H), 2.28 (s, 3H), 1.48 (d, *J* = 6.5 Hz, 3H); ^13^C NMR (CDCl_3_, 125 MHz) δ 158.3, 151.5, 146.8, 137.6, 134.1, 133.7, 133.3, 130.4, 128.6, 126.3, 126.2, 126.0, 125.7, 124.1, 122.8, 121.3, 118.6, 117.8, 55.8, 37.0, 20.0, 19.99, 19.3; DART-HRMS found 390.1604 [M+H]^+^, calcd for C_23_H_24_N_3_OS 390.1640.

Scheme 26. Synthesis of JR-AB2-025

**1-(3,4-Dimethylphenyl)-3-ethyl-1-(5-methyl-4,5-dihydrothiazol-2-yl)urea, JR-AB2-025.** A toluene solution of *N*-(3,4-dimethylphenyl)-5-methyl-4,5-dihydrothiazol-2-amine (**3**, 220 mg, 1.0 mmol, 1.0 eq) and ethyl isocyanate (0.079 mL, 1.0 mmol, 1.0 eq) was refluxed for 1 h under argon gas atmosphere. After the completion of reaction, the mixture was concentrated in vacuo. The residue was purified by flash column chromatography over silica gel (hexane/ethyl acetate, 5:1, v/v) to afford the desired product **JR-AB2-025** (270 mg, 93%) as white solid: Rf = 0.7 (hexane/ethyl acetate, 2:1, v/v); mp 47-49 ^o^C; ^1^H NMR (CDCl_3_, 500 MHz) δ 9.53 (s, 1H), 7.08 (d, *J* = 8.0 Hz, 1H), 6.73 (d, *J* = 2.0 Hz, 1H), 6.68 (dd, *J* = 7.5, 2.0 Hz, 1H), 4.37 (dd, *J* = 11.5, 6.5 Hz, 1H), 3.87 (dd, *J* = 11.5, 7.0 Hz, 1H), 3.62 (sext, *J* = 6.5 Hz, 1H), 3.35 (qd, *J* = 7.5, 5.5 Hz, 2H), 2.25 (s, 3H), 2.24 (s, 3H), 1.40 (d, *J* = 7.0 Hz, 3H), 1.18 (t, *J* = 7.0 Hz, 3H); ^13^C NMR (CDCl_3_, 125 MHz) δ 157.6, 153.8, 147.6, 137.4, 132.8, 130.2, 122.7, 118.5, 55.7, 36.9, 35.0, 19.93, 19.91, 19.3, 15.1; DART-HRMS found 292.1476 [M+H]^+^, calcd for C_15_H_22_N_3_OS 292.1484.

Scheme 27. Synthesis of JR-AB2-026

**3-(tert-butyl)-1-(3,4-dimethylphenyl)-1-(5-methyl-4,5-dihydrothiazol-2-yl)urea, JR-AB2-026.** A toluene solution of *N*-(3,4-dimethylphenyl)-5-methyl-4,5-dihydrothiazol-2-amine (**3**, 220 mg, 1.0 mmol, 1.0 eq) and tert-butyl isocyanate (0.114 mL, 1.0 mmol, 1.0 eq) was refluxed for 1 h under argon gas atmosphere. After the completion of reaction, the mixture was concentrated in vacuo. The residue was purified by flash column chromatography over silica gel (hexane/ethyl acetate, 5:1, v/v) to afford the desired product **JR-AB2-026** (240 mg, 75%) as yellow solid: Rf = 0.5 (hexane/ethyl acetate, 2:1, v/v); mp 75-77 ^o^C; ^1^H NMR (CDCl_3_, 500 MHz) δ 9.69 (s, 1H), 7.08 (d, *J* = 8.0 Hz, 1H), 6.74 (d, *J* = 2.0 Hz, 1H), 6.69 (dd, *J* = 8.0, 2.0 Hz, 1H), 4.35 (dd, *J* = 11.5, 6.5 Hz, 1H), 3.84 (dd, *J* = 11.5, 6.5 Hz, 1H), 3.60 (sext, *J* = 6.5 Hz, 1H), 2.25 (s, 3H), 2.23 (s, 3H), 1.39 (d, *J* = 6.5 Hz, 3H), 1.38 (s, 9H); ^13^C NMR (CDCl_3_, 125 MHz) δ 157.5, 152.4, 147.5, 137.4, 132.7, 130.1, 122.7, 118.5, 55.4, 50.7, 36.6, 29.0, 19.94, 19.92, 19.2; DART-HRMS found 320.1790 [M+H]^+^, calcd for C_17_H_26_N_3_OS 320.1797.

Scheme 28. Synthesis of JR-AB2-027

**2-(3,4-Dichlorophenyl)-N-(3,4-dimethylphenyl)-N-(5-methyl-4,5-dihydrothiazol-2-yl)acetamide, JR-AB2-027.** To a dichloromethane solution of *N*-(3,4-dimethylphenyl)-5-methyl-4,5-dihydrothiazol-2-amine (**3**, 220 mg, 1.0 mmol, 1.0 eq), 3,4-dichlorophenylacetic acid (226 mg, 1.1 mmol, 1.1 eq), EDC HCl (211 mg, 1.1 mmol, 1.1 eq) and HOBT hydrate <14% (175 mg, 1.1 mmol, 1.1 eq) was added diisopropylethylamine (0.37 mL, 2.1 mmol, 2.1 eq) at room temperature. The mixture was stirred for 18 h. After the completion of reaction, the mixture was concentrated in vacuo, and the residue was diluted with ethyl acetate (100 mL) and washed with water (30 mL). The organic layer was dehydrated with brine and MgSO_4_ and then concentrated in vacuo. The residue was purified by flash column chromatography over silica gel (hexane/ethyl acetate, 5:1, v/v) to afford the desired product **JR-AB2-027** (340 mg, 83%) as pale yellow solid: Rf = 0.85 (hexane/ethyl acetate, 2:1, v/v); mp 68-70 ^o^C; ^1^H NMR (CDCl_3_, 500 MHz) δ 7.42 (d, *J* = 2.0 Hz, 1H), 7.37 (d, *J* = 8.5 Hz, 1H), 7.14 (dd, *J* = 8.0, 2.0 Hz, 1H), 7.10 (d, *J* = 8.0 Hz, 1H), 6.66 (s, 1H), 6.63 (dd, *J* = 8.0, 2.0 Hz, 1H), 4.41 (d, *J* = 15.5 Hz, 1H), 4.39 (d, *J* = 15.5 Hz, 1H), 4.34 (dd, *J* = 11.5, 6.5 Hz, 1H), 3.78 (dd, *J* = 11.5, 7.0 Hz, 1H), 3.63 (sext, *J* = 6.5 Hz, 1H), 2.26 (s, 3H), 2.24 (s, 3H), 1.37 (d, *J* = 7.0 Hz, 3H); ^13^C NMR (CDCl_3_, 125 MHz) δ 170.5, 154.5, 147.7, 137.6, 135.2, 132.9, 132.2, 131.8, 130.9, 130.3, 130.2, 129.2, 121.9, 117.6, 55.2, 42.6, 37.3, 19.95, 19.94, 19.3; DART-HRMS found 407.0723 [M+H]^+^, calcd for C_20_H_21_Cl_2_N_2_OS 407.0752.

Scheme 29. Synthesis of JR-AB2-028

**3,4-Dichlorophenyl (3,4-dimethylphenyl)(5-methyl-4,5-dihydrothiazol-2-yl)carbamate, JR-AB2-028.** (11) To a dichloromethane solution (5 mL) of triphosgene (119 mg, 0.4 mmol, 0.4 eq) was added 3,4-dichlorophenol (163 mg, 1.0 mmol, 1.0 eq) in dichloromethane (3 mL) and triethylamine (0.28 mL. 2.2 mmol, 2.2 eq) dropwise at 0 ^o^C. After 10 min stirring at room temperature, then *N*-(3,4-dimethylphenyl)-5-methyl-4,5-dihydrothiazol-2-amine (**3**, 198 mg, 0.9 mmol, 0.9 eq) in dichloromethane (3 mL) was added and the mixture was stirred 2 h at 0 ^o^C. The mixture was concentrated in vacuo, and the residue was diluted with ethyl acetate (100 mL) and washed with aq. NaHCO_3_ solution (30 mL). The organic layer was dehydrated with brine and MgSO_4_ and then concentrated in vacuo. The residue was purified by flash column chromatography over silica gel (hexane/ethyl acetate, 10:1, v/v) to afford the desired product **JR-AB2-028** (208 mg, 51%) as paly yellow oil: Rf = 0.6 (hexane/ethyl acetate, 5:1, v/v); ^1^H NMR (CDCl_3_, 500 MHz) δ 7.44 (d, *J* = 9.0 Hz, 1H), 7.39 (d, *J* = 3.0 Hz, 1H), 7.12 (dd, *J* = 9.0, 3.0 Hz, 1H), 7.07 (d, *J* = 7.5 Hz, 1H), 6.75 (d, *J* = 2.0 Hz, 1H), 6.69 (dd, *J* = 8.0, 2.0 Hz, 1H), 4.38 (dd, *J* = 11.0, 6.0 Hz, 1H), 3.84 (dd, *J* = 11.0, 6.5 Hz, 1H), 3.74 (sext, *J* = 6.5 Hz, 1H), 2.24 (s, 3H), 2.23 (s, 3H), 1.45 (d, *J* = 6.5 Hz, 3H); ^13^C NMR (CDCl_3_, 125 MHz) δ 154.9, 153.3, 149.2, 148.5, 137.3, 132.9, 132.7, 130.6, 130.1, 124.0, 121.9, 121.3, 117.6, 115.3, 56.4, 37.7, 19.9, 19.6, 19.3; DART-HRMS found 409.1515 [M+H]^+^, calcd for C_19_H_19_Cl_2_N_2_O_2_S 409.0544.

Scheme 30. Synthesis of JR-AB2-029

**1-(3,4-Dichlorophenyl)-3-(3,4-dimethylphenyl)-1-methyl-3-(5-methyl-4,5-dihydrothiazol-2-yl)urea, JR-AB2-029.** To a toluene solution of 3-(3,4-dichlorophenyl)-1-(3,4-dimethylphenyl)-1-(5-methyl-4,5-dihydrothiazol-2-yl)urea (**JR-AB2-000**, 102 mg, 0.25 mmol, 1.0 eq), potassium hydroxide (70 mg, 1.25 mmol, 5.0 eq) and tetrabutylammonium bromide (8 mg, 0.025 mmol, 0.1 eq) was added iodomethane (0.078 mL, 1.25 mmol, 5.0 eq) at room temperature. The mixture was stirred for 48 h. After the completion of reaction, the solid was filtered and washed with ethyl acetate. The filtrate was concentrated in vacuo. The residue was purified by flash column chromatography over silica gel (hexane/ethyl acetate, 2:1, v/v) to afford the desired product **JR-AB2-029** (85 mg, 80%) as white powder: Rf = 0.3 (hexane/ethyl acetate, 2:1, v/v); mp 112-113 ^o^C; ^1^H NMR (DMSO-d6, 500 MHz) δ 7.63 (d, *J* = 2.5 Hz, 1H), 7.59 (d, *J* = 8.5 Hz, 1H), 7.31 (dd, *J* = 8.5, 2.5 Hz, 1H), 6.90 (d, *J* = 8.0 Hz, 1H), 6.09 (dd, *J* = 7.5, 2.5 Hz, 1H), 5.95 (s, 1H), 4.01-3.94 (m, 1H), 3.68 (sext, *J* = 6.5 Hz, 1H), 3.58-3.53 (m, 1H), 3.29 (s, 3H), 2.09 (s, 3H), 2.09 (s, 3H), 1.29 (d, *J* = 6.5 Hz, 3H); ^13^C NMR (DMSO-d6, 125 MHz) δ 154.6, 154.0, 148.6, 144.5, 136.9, 131.5, 131.2, 130.5, 130.1, 129.4, 128.7, 125.8, 122.2, 118.0, 56.2, 38.0, 21.5, 20.0, 19.2, 14.0; DART-HRMS found 422.0828 [M+H]^+^, calcd for C_20_H_22_Cl_2_N_3_OS 422.0861.

Scheme 31. Synthesis of Compound JR-AB2-030

**1-(4-Bromophenyl)-3-(2-hydroxypropyl)thiourea, 30.** (4) To an acetone (20 mL) solution of 4-bromophenyl isothiocyanate (428 mg, 2.0 mmol, 1.0 eq) was added *DL*-1-amino-2-propanol (0.2 mL, 2.6 mmol, 1.3 eq) dropwise at room temperature. The mixture was refluxed for 2 h, and then concentrated in vacuo. The residue was purified by flash column chromatography over silica gel (hexane/ethyl acetate, 5:1 🡪 1:1, v/v) to afford the desired product **30** (485 mg, 84%) as brown oil: Rf = 0.1 (hexane/ethyl acetate, 5:1, v/v).

***N*-(4-Bromophenyl)-5-methyl-4,5-dihydrothiazol-2-amine, 31.** (4) To an ethanol (10 mL) solution of 1-(4-bromophenyl)-3-(2-hydroxypropyl)thiourea (**30**, 290 mg, 1.0 mmol, 1.0 eq) was added conc. HCl (5 mL) at room temperature, and the mixture was refluxed for 4 h. After the completion of reaction, the mixture was diluted with ethyl acetate (100 mL) and quenched with aq. NaHCO_3_ to make HCl salt to free form. The organic layer was dehydrated with brine and MgSO_4_ and then concentrated in vacuo. The residue was purified by flash column chromatography over silica gel (hexane/ethyl acetate, 2:1, v/v) to afford the desired product **31** (180 mg, 66%) as white powder: Rf = 0.5 (hexane/ethyl acetate, 1:1, v/v); ^1^H NMR (CDCl_3_, 500 MHz) δ 7.37 (d, *J* = 8.5 Hz, 1H), 6.95 (d, *J* = 8.5 Hz, 1H), 3.89 (sext, *J* = 6.5 Hz, 1H), 3.81 (dd, *J* = 10.5, 6.5 Hz, 1H), 3.38 (dd, *J* = 10.0, 6.5 Hz, 1H), 1.44 (d, *J* = 6.5 Hz, 3H); ^13^C NMR (CDCl_3_, 125 MHz) δ 162.1, 147.4, 132.0, 123.2, 116.1, 55.7, 43.2, 20.3.

**1-(4-Bromophenyl)-3-(3,4-dichlorophenyl)-1-(5-methyl-4,5-dihydrothiazol-2-yl)urea, JR-AB2-030.** A toluene (10 mL) solution of *N*-(4-bromophenyl)-5-methyl-4,5-dihydrothiazol-2-amine (**31**, 135 mg, 0.5 mmol, 1.0 eq) and 3,4-dichlorophenyl isocyanate (94 mg, 0.5 mmol, 1.0 eq) was refluxed for 1 h. After the completion of reaction, the mixture was concentrated in vacuo, and the residue was crystallized using ethyl acetated and hexane to afford the desired product **JR-AB2-030** (220 mg, 96%) as white powder: Rf = 0.8 (hexane/ethyl acetate, 2:1, v/v); mp 155-157 ^o^C; ^1^H NMR (CDCl_3_, 500 MHz) δ 12.0 (s, 1H), 7.72 (d, *J* = 2.0 Hz, 1H), 7.48 (d, *J* = 8.5 Hz, 2H), 7.34 (d, *J* = 9.0 Hz, 1H), 7.30 (dd, *J* = 9.0, 2.0 Hz, 1H), 6.88 (d, *J* = 8.5 Hz, 2H), 4.45 (dd, *J* = 11.5, 6.5 Hz, 1H), 3.98 (dd, *J* = 11.5, 7.0 Hz, 1H), 3.73 (sext, *J* = 6.5 Hz, 1H), 1.47 (d, *J* = 6.5 Hz, 3H); ^13^C NMR (CDCl_3_, 125 MHz) δ 159.3, 150.6, 147.7, 137.6, 132.7, 132.4, 130.4, 126.8, 123.3, 121.5, 119.2, 118.3, 55.7, 37.1, 20.1; DART-HRMS found 457.9460 [M+H]^+^, calcd for C_17_H_14_BrCl_2_N_3_OS 457.9496.

References

1. Kaiser C., Michaelis A., Mitchell C. Methods in yeast genetics: a Cold Spring Harbor laboratory course manual, 1994 ed. Cold Spring Harbor Laboratory Press, Cold Spring Harbor, N.Y.

2. James P., Haliaday J., Craig E. Genomic libraries and a host strained designed for highly efficient two-hybrid selection in yeast. Genetics **1996**; 144: 1425-1436.

3. Kamada Y., Fujioka Y., Suzuki N., Inagaki F., Wullschleger S., Loewith R., Hall M., Ohsumi Y. Tor2 directly phosphorylates the AGC kinase Ypk2 to regulate actin polarization. Mol. Cell Biol. **2005**; 16: 7239-7248.

4. Goodyer C., Chinje E., Jaffar M., Stratford I., Threadgill M. [Synthesis of *N*-benzyl- and *N*-phenyl-2-amino-4,5-dihydrothiazoles and thioureas and evaluation as modulators of the isoforms of nitric oxide synthase](http://www.sciencedirect.com/science/article/pii/S0968089603004516). Bioorg. Med. Chem. **2003**; 11: 4189-4206.

5. [Kasai](http://pubs.acs.org/author/Kasai%2C+Shizuo) S., [Kamata](http://pubs.acs.org/author/Kamata%2C+Makoto) M., [Masada](http://pubs.acs.org/author/Masada%2C+Shinichi) S., [Kunitomo](http://pubs.acs.org/author/Kunitomo%2C+Jun) J., [Kamaura](http://pubs.acs.org/author/Kamaura%2C+Masahiro) M., [Okawa](http://pubs.acs.org/author/Okawa%2C+Tomohiro) T., [Takami](http://pubs.acs.org/author/Takami%2C+Kazuaki) K., [Ogino](http://pubs.acs.org/author/Ogino%2C+Hitomi) H., [Nakano](http://pubs.acs.org/author/Nakano%2C+Yoshihide) Y., [Ashina](http://pubs.acs.org/author/Ashina%2C+Shuntarou) S., [Watanabe](http://pubs.acs.org/author/Watanabe%2C+Kaoru) K., [Kaisho](http://pubs.acs.org/author/Kaisho%2C+Tomoko) T., [Imai](http://pubs.acs.org/author/Imai%2C+Yumi+N) Y., [Ryu](http://pubs.acs.org/author/Ryu%2C+Sunghi) S., [Nakayama](http://pubs.acs.org/author/Nakayama%2C+Masaharu) M., [Nagisa](http://pubs.acs.org/author/Nagisa%2C+Yasutaka) Y., [Takekawa](http://pubs.acs.org/author/Takekawa%2C+Shiro) S., [Kato](http://pubs.acs.org/author/Kato%2C+Koki) K., [Murata](http://pubs.acs.org/author/Murata%2C+Toshiki) T., [Suzuki](http://pubs.acs.org/author/Suzuki%2C+Nobuhiro) N., [Ishihara](http://pubs.acs.org/author/Ishihara%2C+Yuji) Y. Synthesis, structure–activity relationship, and pharmacological studies of novel melanin-concentrating hormone receptor 1 antagonists 3-aminomethylquinolines: Reducing human ether-a-go-go-related gene (hERG) associated liabilities. J. Med. Chem. **2012**; 55: 4336-4351.

6. Liu D., Tian Z., Yan Z., Wu L., Ma Y., Wang Q., Liu W., Zhou H., Yang C. [Design, synthesis and evaluation of 1,2-benzisothiazol-3-one derivatives as potent caspase-3 inhibitors](http://www.sciencedirect.com/science/article/pii/S0968089613002976). Bioorg. Med. Chem. **2013**; 21: 2960-2967.

7. Mihovilovic M., Schnuerch M., Waldner, B., Hilber K. Novel thiazolamine derivates as differentiation accelerators. PCT Int. Appl. WO2012040754, 05 Apr **2012**.

8. Yao R., Liu H., Wu Y., Cai M. Ligand- and solvent-free synthesis of 2-aminobenzothiazoles by copper-catalyzed tandem reaction of 2-haloanilines with isothiocyanates. Appl. Organometal. Chem. **2013**; 27: 109-113.

9. [Huang](http://pubs.rsc.org/en/results?searchtext=Author%3AXiaomei%20Huang) X., [Xu](http://pubs.rsc.org/en/results?searchtext=Author%3AShuguang%20Xu) S., [Tan](http://pubs.rsc.org/en/results?searchtext=Author%3AQitao%20Tan) Q., [Gao](http://pubs.rsc.org/en/results?searchtext=Author%3AMingchun%20Gao) M., [Li](http://pubs.rsc.org/en/results?searchtext=Author%3AMinjie%20Li) M., [Xu](http://pubs.rsc.org/en/results?searchtext=Author%3ABin%20Xu), B. A copper-mediated tandem reaction through isocyanide insertion into N–H bonds: efficient access to unsymmetrical tetrasubstituted ureas. Chem. Comm. **2014**; 50: 1465-1468.

10. Wang X., Jang H.-Y. All purpose copper catalyst for coupling of ammonium salts and 1^o^ and 2^o^ amine with boronic acid. Bull. Korean Chem. Soc. **2012**; 33: 1785-1787.

11. Majer P., Randad R. A safe and efficient method for preparation of *N,N’*-unsymmetrically disubstituted ureas utilizing triophosgene. J. Org. Chem. **1994**; 59: 1937-1938.
